# Case Report: Representational geometry of multi-digit coordination in the macaque grasping network for intracortical BCIs

**DOI:** 10.3389/fnins.2026.1933898

**Published:** 2026-10-27

**Authors:** Wei-An Sheng, Andres Agudelo-Toro, Benjamin Dann, Hansjörg Scherberger

**Affiliations:** 1 Deutsches Primatenzentrum GmbH, Göttingen, Germany; 2 Collaborative Research Center CRC 1690 “Disease Mechanisms and Functional Restoration of Sensory and Motor Systems”, University of Göttingen, Göttingen, Germany; 3 Faculty of Biology and Psychology, University of Göttingen, Göttingen, Germany; 4 Else Kröner Fresenius Center for Optogenetic Therapies, University Medical Center, Göttingen, Germany

**Keywords:** brain-computer interfaces, case report, finger movement representation, grasping circuit, intracortical recording, macaque, motor cortex, neural population dynamics

## Abstract

**Introduction:**

Intracortical brain-computer interfaces (BCIs) hold considerable promise for restoring dexterous hand function in individuals with severe paralysis or limb loss. However, reliable control of coordinated multi-digit movements remains challenging because the cortical representation of finger coordination is not fully understood. Whether this representation is shared across the cortical areas that contribute to grasping, or instead varies from one area to another, remains unclear.

**Case report:**

We report the surgical implantation and neural recordings from a male rhesus macaque implanted with six floating microelectrode arrays (192 channels) across three nodes of the cortical grasping network: the anterior intraparietal area (AIP), ventral premotor cortex (area F5), and primary motor cortex (M1). The subject was trained to perform individual and combined finger flexions using a microswitch-based manipulandum. To our knowledge, this is the first study to record intracortical population activity simultaneously from these three cortical areas during coordinated multi-digit movements.

**Observations:**

Population activity in AIP, F5, and M1 diverged into distinct, movement-specific trajectories. The number of independent activity patterns needed to capture most of these movement-related differences was larger in F5 than in AIP or M1. Alignment and reconstruction analyses additionally showed descriptive area-specific patterns in how closely combined-movement activity resembled a simple combination of the two single-finger activity patterns it comprises. This resemblance was generally limited across a range of dimensionalities. Error-trial analyses showed complementary within-area temporal patterns: early AIP activity preferentially reflected the instructed movement, whereas M1 activity near erroneous presses reflected the movement produced.

**Conclusion:**

In this animal, condition-dependent dimensionality was significantly higher in F5 than in AIP or M1, while alignment and reconstruction analyses showed descriptive area-specific patterns in how closely combined-finger activity resembled a simple combination of single-finger activity. For brain-computer interfaces, these observations raise the hypothesis that whether single-finger training generalizes to combined presses may depend on which cortical area a decoder reads from, not only on how it is built. Shared low-dimensional structure could still support a small, easy-to-calibrate signal set in some areas, while area-specific structure could limit generalization in others. These possibilities require direct comparisons of decoder performance and calibration strategies across areas, as well as replication beyond this single subject.

## 1 Introduction

The primate hand is a highly versatile and complex motor organ, capable of rapidly switching among a rich repertoire of prehensile and non-prehensile configurations (Feix et al., 2016). Disruption of hand function caused by neurological disorders or spinal cord injury severely limits functional independence, making restoration of hand and arm function a major clinical priority (Anderson, 2004). Intracortical brain-computer interfaces (BCIs) provide a direct route toward restoring dexterity by translating neural population activity into control signals for robotic prosthetic hands or functional electrical stimulation systems (Carmena et al., 2003; Hochberg et al., 2012; Collinger et al., 2013; Agudelo-Toro et al., 2024). While high-density microelectrode arrays and anthropomorphic prostheses have advanced significantly (Piazza et al., 2019), current clinical BCI decoders are still largely restricted to simple, gross grasping configurations and cannot yet reproduce the fine, coordinated multi-digit controls of the native hand (Nason et al., 2021).

Although neural decoding during object grasping has successfully reconstructed complex hand kinematics (Vargas-Irwin et al., 2010; Menz et al., 2015), finger movements during grasping are highly correlated (Mason et al., 2001), providing limited insight into the neural control of individuated and coordinated finger movements required for behaviors such as typing, button pressing, or tool manipulation. A central unresolved question is whether neural activity associated with simultaneous multi-digit movements can be predicted from the representations of individual digits or whether combined finger movements constitute distinct neural population states. It remains similarly unclear whether any such distinction is uniform across the cortical areas that contribute to grasping, or instead varies in both the timing and the structure of the underlying activity. This distinction has important implications for BCI decoder design: if neural representations are compositional, decoders could be calibrated using isolated single-digit movements and generalized to novel finger combinations, substantially reducing calibration time and user effort (Choi et al., 2018); if combined movements are represented non-linearly, decoders must instead explicitly learn these movement configurations using more sophisticated state-dependent or machine-learning approaches. Even when combined-movement activity cannot be fully predicted from single-finger activity, some single-finger-related structure can still be present within it.

On the one hand, studies have shown that neural activity in primary motor cortex (M1) can be approximated by linear superposition of single-finger representations, allowing decoders trained on isolated movements to generalize to combined finger movements (Baker et al., 2010; Choi et al., 2018; Nason et al., 2021). On the other hand, studies have reported non-linearities, indicating that combined movements recruit distinct population representations that cannot be predicted from isolated finger movements alone (Ben Hamed et al., 2007; Shah et al., 2025). Multi-finger representation has also been shown to be non-linear more broadly, characterized by magnitude normalization and context-dependent tuning shifts in human premotor cortex (Shah et al., 2025), rich tactile interactions in sensorimotor cortex (Arbuckle et al., 2022), and non-linear biomechanical coupling (Schieber, 1991; Häger-Ross and Schieber, 2000; Schieber and Santello, 2004).

Research on combined-digit representations has historically focused almost exclusively on primary motor cortex, although the representation of grasping movements and decoding ability have also been explored in posterior parietal and premotor cortex (Lehmann and Scherberger, 2013; Schaffelhofer et al., 2015; Guan et al., 2023; Agudelo-Toro et al., 2024); a multi-area investigation of combined-digit representations across the grasping circuit is still missing. A notable exception is Yokoi et al. (2018), who showed that sequential multi-finger movements recruit sequence-specific representations in upstream premotor and parietal areas, in contrast to linear execution patterns in primary motor cortex; however, this study focused on sequences and did not address simultaneous execution of two fingers.

Here, we simultaneously recorded from AIP, F5, and M1 while a macaque performed individual and combined finger movements using a microswitch-based manipulandum, targeting three key areas of the primate grasping circuit (Luppino et al., 1999; Rizzolatti et al., 1988; Schieber and Hibbard, 1993). We addressed these questions by directly comparing AIP, F5, and M1: whether combined-movement activity was captured by the activity patterns of its two single-finger constituents, and whether those patterns could reconstruct it. Combined movements were generally not reducible to this simple structure across a range of dimensionalities, and the degree to which they were reducible differed across the three areas. Error trials additionally revealed a difference in timing across these same areas: AIP activity remained linked to the instructed movement for longer, while M1 activity shifted earlier toward the movement actually produced. The animal had acquired the task sufficiently for planned recording and real-time decoding, although performance was not fully optimized; the experiment ended for reasons unrelated to task performance, preventing further recording. This single-subject dataset therefore provides a simultaneous, descriptive comparison of how three interconnected cortical areas represent coordinated finger movements.

## 2 Case description

One male rhesus macaque (*Macaca mulatta*, 10.5 kg) was implanted with intracortical microelectrode arrays and was subsequently trained in an individuated and combined finger-movement task (Figures 1a, b, Supplementary methods). The subject received six 32-channel floating microelectrode arrays, providing 192 channels across three nodes of the cortical grasping circuit (Luppino et al., 1999; Rizzolatti et al., 1988; Schieber and Hibbard, 1993): the anterior intraparietal area (AIP, lateral end of the intraparietal sulcus), the ventral premotor area F5 (posterior bank of the arcuate sulcus), and the primary motor cortex (M1, anterior bank of the central sulcus) (Figures 1c, d). These three areas were selected based on the available simultaneous implant configuration together with their established reciprocal anatomical and functional connectivity within the dorsolateral grasping circuit, in which AIP and F5 interact with M1 during grasp-related sensorimotor processing, without implying that AIP is the only, or uniquely primary, parietal area contributing to grasping, as other posterior parietal regions such as area V6A are also involved. Electrode lengths ranged from 1.5 to 7.1 mm to match the depth profile of each implantation site. All animal housing, care, and experimental procedures complied with German and European animal-welfare laws and were approved by the local veterinary authority (see Ethics Statement).

**Figure 1:**
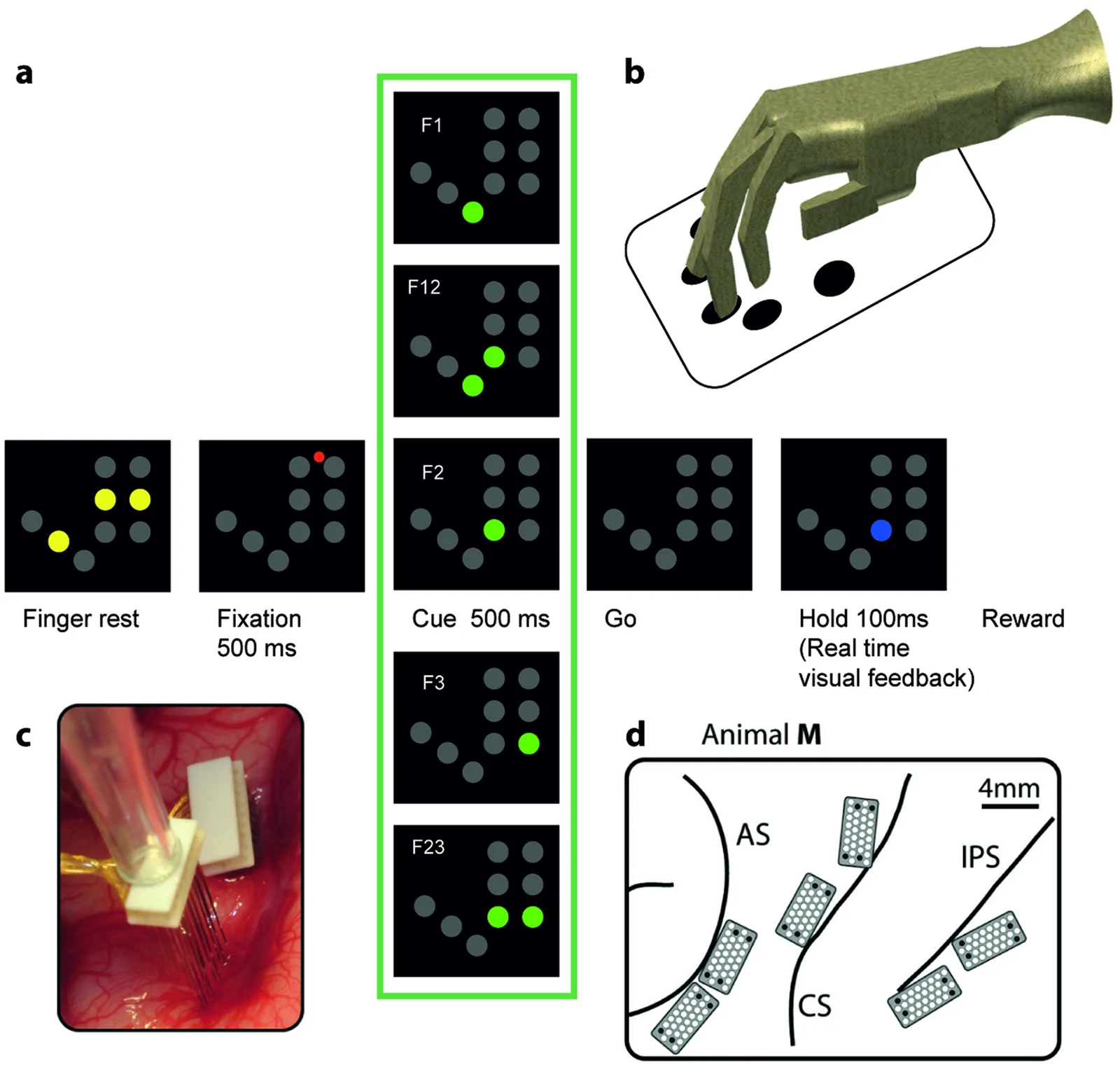
Experimental setup, task paradigm and array implantation. **(a)** Task paradigm. The gray dots mark the potential positions for cues and were not shown to the monkey. The positions of the dots mimic the thumb (F1), index (F2) and middle (F3) finger of the right hand. There were three levels for each finger, where the second level showed the finger rest positions (yellow) and the third level showed the movement cue (green) and real-time feedback of the button press (blue). The first level was not used in this study. The five cues used for single and combined movements are shown in the middle column (surrounded by a green rectangle). **(b)** Illustration of the microswitch manipulandum for detecting flexion movements of the right hand used in this study. **(c)** Photograph of the floating microelectrode array (FMA) insertion procedure. **(d)** Anatomical locations of the six FMAs implanted simultaneously in the left hemisphere of monkey M in areas AIP, F5, and M1. Each array consisted of a 4x9 matrix with two ground and two reference electrodes (black), and 32 recording channels (white). Electrode length increased toward the sulcus from 1.5 mm to 7.1 mm. IPS: intraparietal sulcus; AS: arcuate sulcus; CS: central sulcus.

The behavioral task required the subject to perform individual and combined finger movements using a custom-designed manipulandum with five microswitches arranged to match the anatomical positions of the fingertips on the right hand (Figures 1a, b). Each trial began when the subject placed the index and middle fingers on touch-sensitive switches, initiating a 500-ms fixation epoch, followed by a 500-ms visual cue (green dots) instructing one of five movement conditions: thumb flexion (F1), index flexion (F2), middle-finger flexion (F3), simultaneous thumb-index flexion (F12), or simultaneous index-middle flexion (F23). Cue offset served as the go signal, upon which the subject flexed the instructed digit or digit pair to activate the corresponding microswitch(es); successful activation maintained for 100 ms triggered a juice reward with visual feedback (blue dots). For combined movements, both microswitches had to be activated within a 100-ms interval to encourage simultaneous rather than sequential finger movements.

Recording began after the animal had acquired the task sufficiently for the planned neural-recording and real-time-decoding experiments, although behavioral performance remained variable and not fully optimized. After six recording sessions, experiments had to be stopped for reasons unrelated to the finger-movement study, preventing further recording and behavioral optimization. The available dataset therefore comprises six planned recording sessions with moderate, condition-dependent behavioral performance.

## 3 Results

### 3.1 Behavioral performance

We first analyzed behavioral performance during the delayed finger-movement task. Across six recording sessions, the subject completed 1,924 of 4,051 trials that reached cue presentation (47.5%; approximately 48%), with pooled condition-specific success rates across the six sessions of 54% for individual thumb flexion (F1), 39% for index flexion (F2), 63% for middle-finger flexion (F3), 32% for combined thumb-index flexion (F12), and 60% for combined index-middle flexion (F23). F12 remained more difficult than F23 across sessions, whereas F23 was relatively high in most sessions except Rec5, and overall performance fluctuated without a clear monotonic increase (Supplementary Figure S1); thus, generic two-digit difficulty alone cannot explain the F12–F23 difference, which may instead reflect digit-specific biomechanics, movement strategy, manipulandum demands, or condition-specific task proficiency. These possibilities cannot be separated without kinematic, force, or EMG measurements. Despite the moderate overall success rate, the subject's performance across all conditions was statistically significant compared to both a nominal 5-choice chance baseline (20%) and a combinatorial 7-choice chance baseline (14.3%). For each movement condition, trials were pooled across the six recording sessions and a one-sided exact binomial test was applied to the total number of successful trials out of all trials that reached cue presentation. All five conditions exceeded both chance baselines (20% baseline: all *p* ≤ 8.78 × 10^−19^; 14.3% baseline: all *p* ≤ 1.29 × 10^−44^; exact *k*, *n*, and *p*-values are reported in Supplementary methods 1.4), indicating that the subject understood the task instructions and engaged in goal-directed planning rather than performing random finger combinations.

To ensure that subsequent neural analyses were restricted to trials representing active task engagement and consistent preparatory states, successful trials also had to satisfy a coordinated double-movement criterion and a reaction-time (RT) criterion excluding the long tails of the RT distributions (Supplementary methods). Of 1,924 task-successful trials, 1,538 remained after the double-movement validity criterion and 1,272 after the historical RT thresholds (the fixed thresholds used in our initial analysis, retained here for comparison with the MAD-based sensitivity analysis described below): RT filtering thus retained 82.7% of the behaviorally valid pre-RT pool, corresponding to a final retention of 66.1% of originally successful trials (212 trials/session). A condition-specific median-plus-3-median-absolute-deviations (MAD) sensitivity analysis retained 95.1% of the pre-RT pool instead (Supplementary Figure S2) and preserved the qualitative conclusions of the population analyses described below. Following thresholding, reaction times were relatively short for single-finger movements (F1: 81 ± 51 ms; F2: 97 ± 58 ms; F3: 83 ± 51 ms), whereas combined movements required substantially longer preparation (F12: 221 ± 83 ms; F23: 203 ± 80 ms). Although the task required precise synchronization of multiple microswitch activations within a 100 ms window, each condition retained, on average, more than 13 correct trials per session.

### 3.2 Single-unit characterization

Following spike sorting (Quiroga et al., 2004; Michaels et al., 2015; Dann et al., 2016), we characterized task-related neural activity across the three recorded regions, recording an average of 63 ± 6.4 units simultaneously in AIP, 72.5 ± 6.0 in F5, and 92.2 ± 10.1 in M1. A high proportion of units exhibited significant task-related modulation (AIP: 74%; F5: 78%; M1: 79%; cluster-based permutation test), with individual neurons displaying broad and heterogeneous tuning across movement conditions (Supplementary Figure S3).

Aligning units by tuning onset revealed area-specific temporal profiles of task-related tuning: AIP showed an earlier numerical increase in the proportion of tuned units after cue onset (Figure 2a), and during the first 250 ms following cue presentation, 12.3% of AIP units were significantly tuned, compared with 8.1% in F5 and 7.5% in M1. An initial pooled unit-level analysis, in which recorded units were pooled across sessions, detected a significant cortical-area effect from approximately 60–220 ms after cue onset (cluster-corrected *p* = 0.007, Supplementary methods). However, because units were nested within recording sessions, we followed this with a direct session-level analysis using the six simultaneous recording sessions as the resampling unit. In these session-level comparisons, neither the early AIP–F5 difference nor the early AIP–M1 difference reached significance (AIP–F5: minimum cluster-corrected *p* = 0.283; AIP–M1: *p* = 0.070, Figure 2b). A significant between-area difference emerged later in the trial, when the proportion of tuned units was lower in AIP than in M1 from approximately 100 ms before to 300 ms after hold onset (cluster-corrected *p* = 0.009, Figure 2b). No significant AIP–F5 cluster was detected. We therefore interpret the earlier AIP rise as a descriptive temporal pattern rather than as a statistically established difference in tuning onset. This temporal pattern is consistent with AIP contributing to the early processing of visuomotor information before preparatory and movement-related activity becomes more prominent in premotor and primary motor cortex. However, because cue identity is coupled to the instructed movement, this correct-trial analysis alone cannot distinguish visual/instruction-related activity from motor intention.

**Figure 2:**
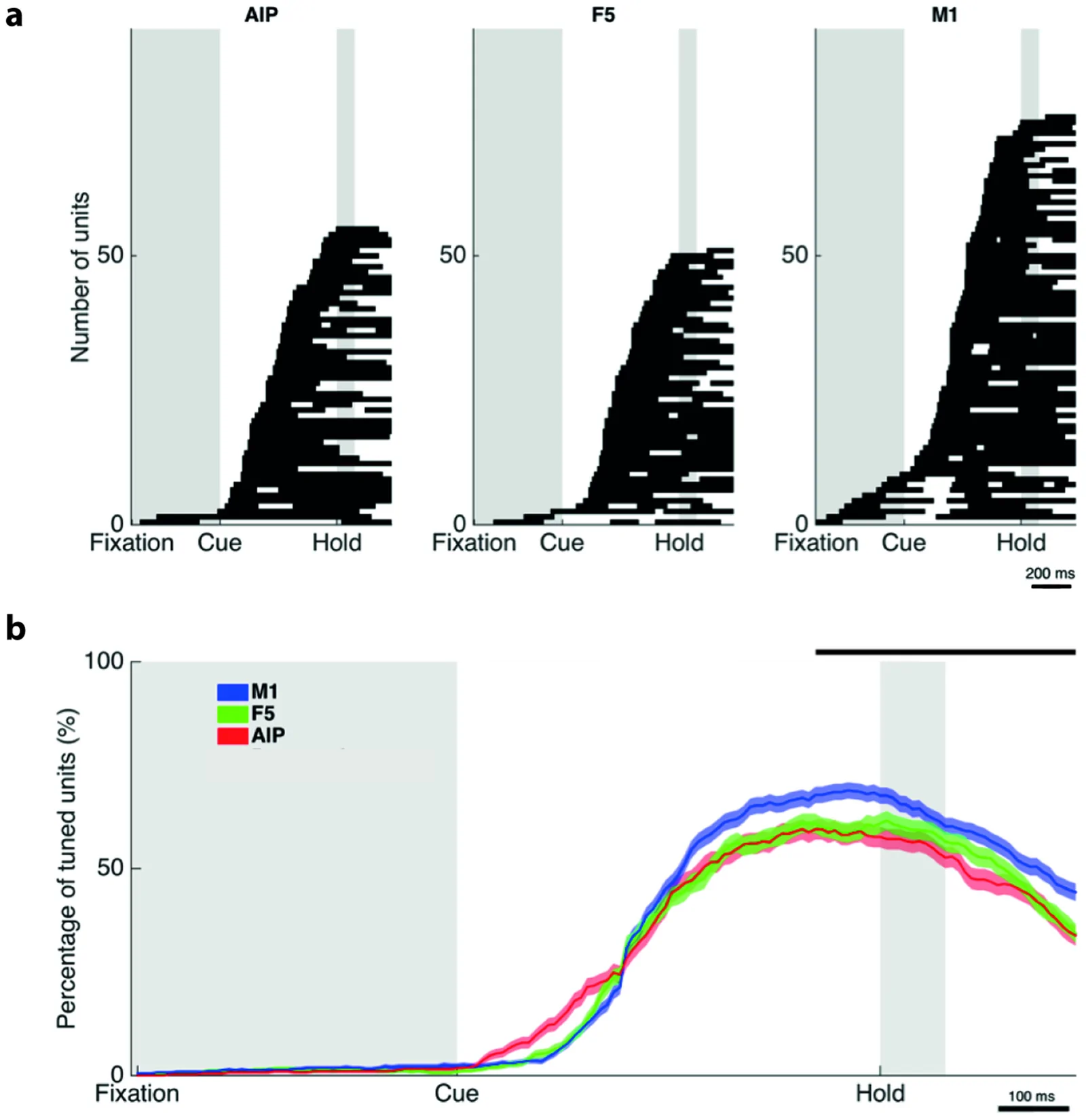
Tuning characteristics of units in areas AIP, F5, and M1 based on the cluster-based permutation test. **(a)** Tuning onset of one example recording. Individual units in each area were vertically aligned by their time of tuning onset, and times of significant tuning are marked as horizontal black bars. Onsets of fixation, cue, and hold epochs are marked. **(b)** Percentages of tuned units in areas AIP, F5, and M1, averaged across six recording sessions, shadows showing standard error. The horizontal significance bar indicates the interval with a significant direct AIP–M1 difference in the session-level cluster permutation analysis (AIP < M1; FWER-corrected *p* = 0.009). No significant AIP–F5 cluster was detected.

We therefore analyzed error trials in which instructed and executed finger movements differed (Supplementary Figure S4, Supplementary methods). After cue onset, AIP and F5 population activity preferentially matched the instructed condition, whereas M1 did not show the same early preference. Around cue offset/go, AIP remained instruction-biased, F5 became intermediate, and M1 shifted toward the executed condition. When aligned to the erroneous microswitch press, M1 preferentially matched the movement actually produced. These results support a graded transition from instructed/visuomotor to execution-related correspondence rather than uniquely identifying early AIP activity as motor intention.

The heterogeneous tuning observed at the single-unit level illustrates the distributed nature of neuronal responses across movement conditions (Schieber and Hibbard, 1993; Poliakov and Schieber, 1999). Representative functional classes, including neurons preferentially responsive to single-finger movements, combined finger movements, and shared movement features, are shown in Supplementary Figure S5. Overall, we found tuning for all five conditions, indicating that the subject reliably distinguished the different finger presses. Together, these findings show that all three cortical areas contain robust but distributed and mixed task-related selectivity, motivating population-level analyses of coordinated finger movements.

### 3.3 Population representation of coordinated finger movements

Because single neurons showed mixed and heterogeneous tuning, we next examined the structure of movement representations at the population level, a framework better suited to characterizing and decoding multi-digit representations (Schieber and Hibbard, 1993; Georgopoulos et al., 1999; Shah et al., 2025).

As an initial measure of representational similarity, we computed pairwise Spearman rank correlations between condition-specific population activity vectors (Supplementary Tables). Although combined movements shared features with their constituent single-finger movements, these relationships were asymmetric: combined index-middle flexion (F23) was positively correlated with middle-finger flexion (F3) across all three areas (AIP: 0.38; F5: 0.37; M1: 0.44), but negatively correlated with index-finger flexion (F2) (AIP: -0.50; F5: -0.33; M1: -0.26). These findings provide a first indication that combined finger movements are not represented as simple averages of their constituent digits but instead involve context-dependent modulation of individual finger representations.

To examine the representational geometry of population activity, we isolated condition-dependent activity by subtracting the across-condition mean response and projected the result into a low-dimensional state space using principal component analysis (PCA). Population trajectories for the five movement conditions followed distinct, largely non-overlapping paths that emerged from a common initial trajectory before diverging into movement-specific structure (Figure 3). In the representative session shown in Figure 3, the condition-dependent activity defined above accounted for 57.9% of total population variance in AIP, 22.9% in F5, and 39.2% in M1 (obtained using dPCA). Within this condition-dependent variance, the first three PCs captured 60.3% in AIP, 48.7% in F5, and 67.1% in M1. Thus, the three-dimensional trajectories visualize a substantial component of the movement-related population structure but do not provide a complete representation of its geometry. To quantify dimensionality more objectively, for each recording session and cortical area we determined the minimum number of PCs required to explain 90% (D90) or 95% (D95) of the condition-dependent variance. Mean D90/D95 values were 11.8/18.2 PCs in AIP, 17.8/24.8 PCs in F5, and 11.7/18.3 PCs in M1 (Supplementary Figure S6). Direct session-level comparisons confirmed that dimensionality was higher in F5 than in both AIP and M1 for D90 (F5–AIP: *p* = 0.0045; F5–M1: *p* = 0.0027) and D95 (F5–AIP: *p* = 0.0051; F5–M1: *p* = 0.0021; all six values FWER-corrected). Thus, describing the differences between movement types required more independent activity patterns in F5 than in AIP or M1.

**Figure 3:**
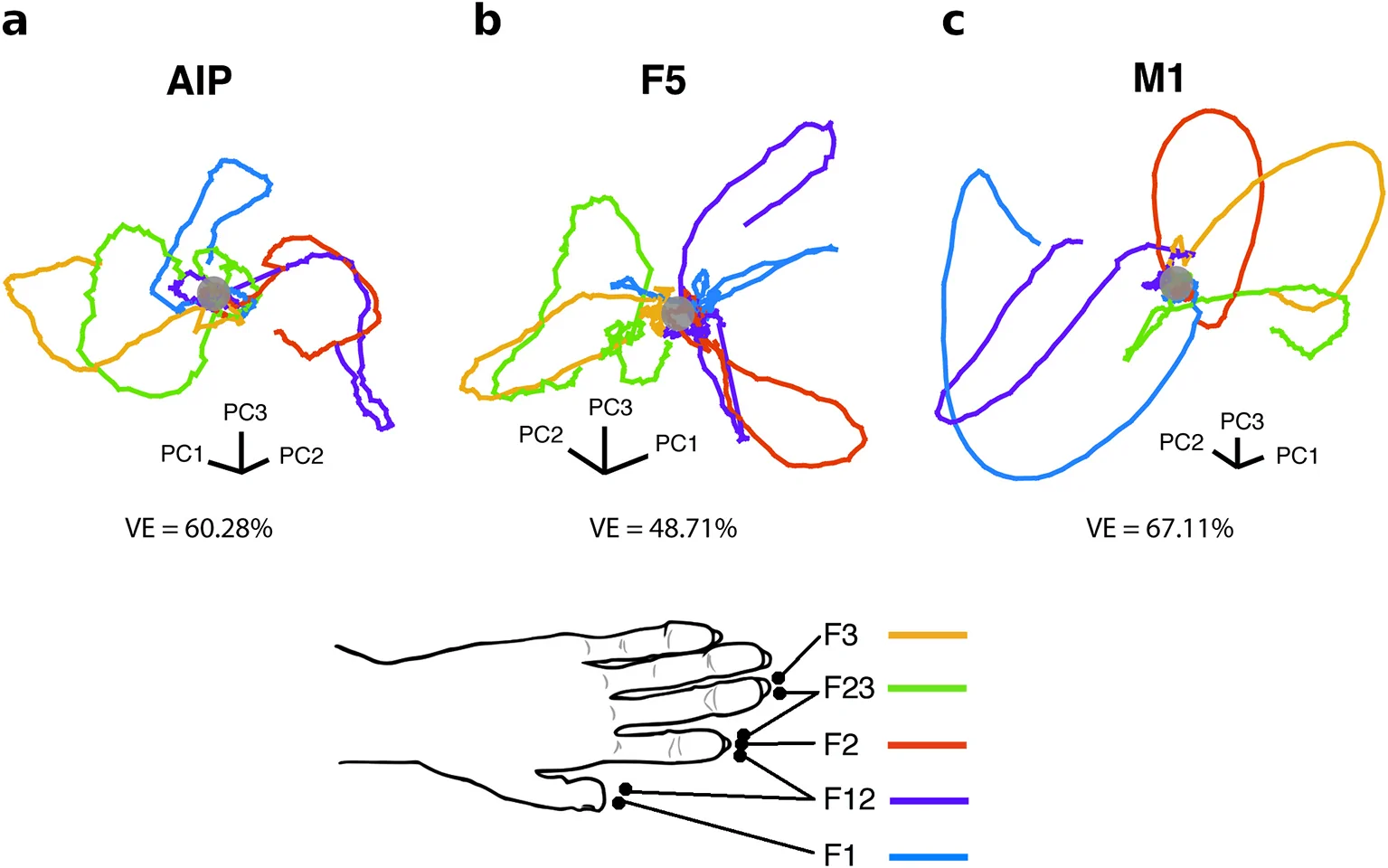
Neural trajectories of the condition-dependent neural activity in the first three principal component (PC) space. **(a–c)** Three-dimensional state-space trajectories of the five movement conditions for area AIP **(a)**, area F5 **(b)**, and area M1 **(c)** from one example recording session. The percentage of variance explained (VE) by the first three PCs is indicated at the bottom. Trajectories of the five finger movement conditions (three single and two combined) are plotted in different colors (color code shown in the schematic at the bottom). Gray circles mark the start of each trial (onset of the fixation epoch).

Following Raposo et al. (2014), we quantified how closely combined-movement activity resembled the activity patterns of its two single-finger constituents (Figure 4). An initial analysis used eight PCs and showed that combined movements were less aligned with this constituent-defined activity space than the constituent positive controls, supporting incomplete capture by constituent geometry. We then repeated the analysis across 3, 5, 8, 10, 12, 15, and 20 PCs and at session-specific D90/D95 (Supplementary Figure S7). Constituent-specific alignment relative to an unrelated movement appeared in some lower-dimensional spaces but weakened as more dimensions were retained and was not consistently present at D90/D95. Thus, combined-movement activity shared some structure with its constituents in a small number of dominant patterns, but was not fully captured by the constituent-defined activity space in AIP, F5, or M1.

**Figure 4:**
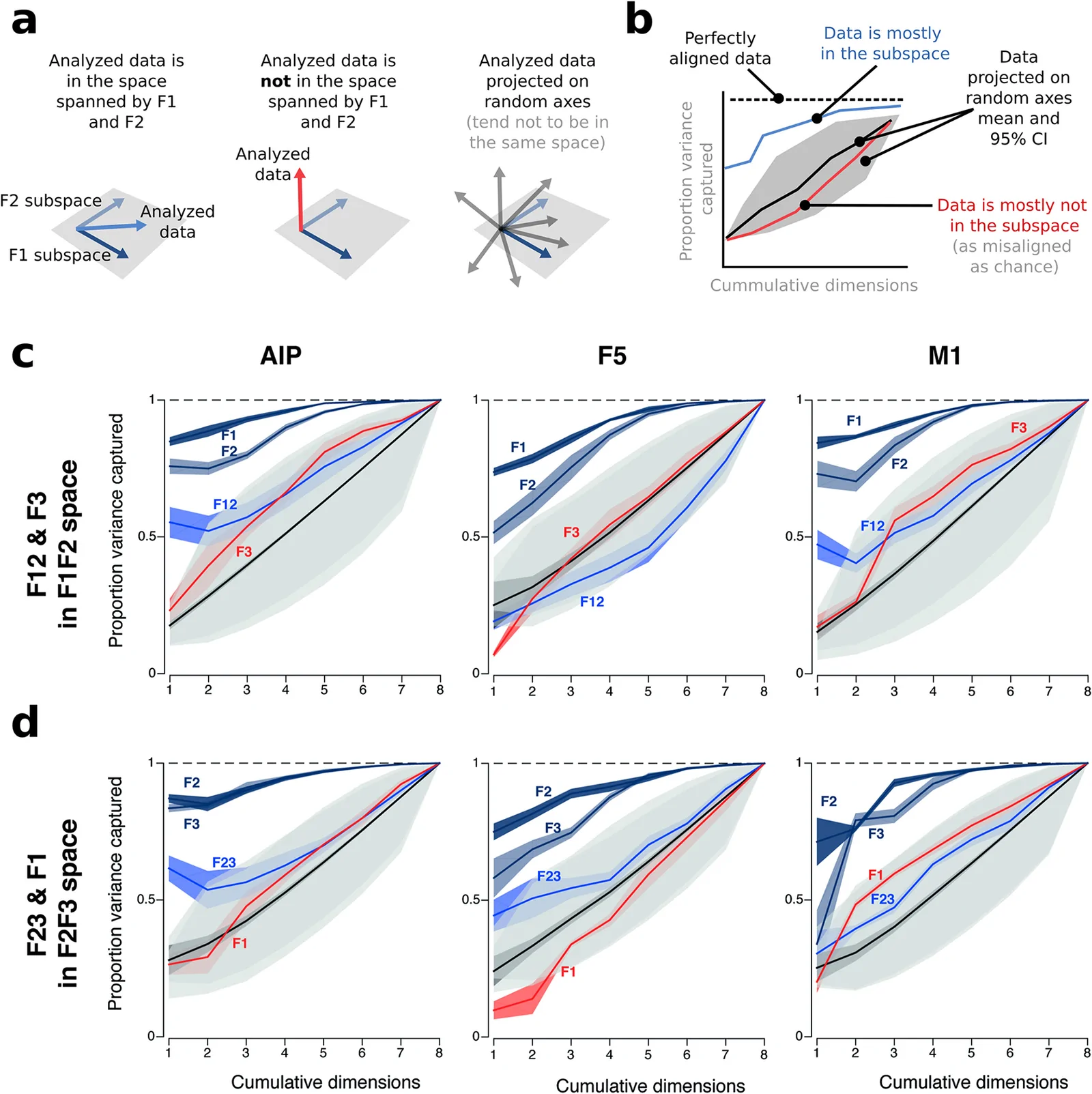
Subspace variance projection analysis for combined and unrelated single finger movements. **(a)** Conceptual schematic of variance projection onto the subspace spanned by two related single finger movements (e.g., *F*_1_ and *F*_2_). **(b)** Schematic of the cumulative variance curves expected for aligned (blue), random/chance [black, with 95% confidence interval (CI) in gray], and misaligned (red) data projections. **(c)** Proportion of variance of the thumb-index combined movement (*F*_12_, blue) and the unrelated control middle finger movement (*F*_3_, red) captured in the reference subspace spanned by the constituent related single finger movements (*F*_1_ and *F*_2_) across areas AIP, F5, and M1. **(d)** Same as in **(c)** for the projection of the index-middle combined movement (*F*_23_, blue) and the unrelated control thumb movement (*F*_1_, red). **(c, d)**, Dark and light teal: positive controls. Black curve: mean chance level. Wide gray shaded regions: 95% CI for the chance distributions of the combined and non-related single movements. Narrow gray shaded region around the black line: standard error (S.E.) of the mean chance projection. Narrow colored shaded regions: S.E. of the respective mean trajectories.

Multiple linear regression provided a complementary test of whether single-finger activity could reconstruct combined-movement activity. An initial analysis used five PCs (Supplementary Figure S8); F1 and F2 predicted F12 with F3 as an unrelated control, and F2 and F3 predicted F23 with F1 as control. Across 3–20 PCs and session-specific D90/D95 (Supplementary Figure S9), reconstruction from single-finger activity was generally incomplete: F23 was not preferentially reconstructed from F2 and F3, particularly in AIP and M1, and F12 showed little constituent-specific reconstruction in AIP and F5, though in M1 F1 and F2 showed numerically stronger reconstruction of F12 than was observed in AIP or F5. This pattern was also maintained under the MAD-based reaction-time criterion. Cross-area differences in the alignment and reconstruction summaries were not subjected to direct between-area statistical tests and are therefore interpreted descriptively. Together, these analyses show a statistically supported difference in condition-dependent dimensionality, with higher dimensionality in F5, alongside descriptive area-specific patterns in how closely combined-movement activity resembles a simple combination of its constituents.

### 3.4 Comparison across cortical areas

Bringing together the single-unit and population-level results above, AIP, F5, and M1 differed from one another through a combination of directly tested and descriptive area-specific patterns rather than forming a single, homogeneous population:

• Population trajectories for the five conditions followed distinct, largely non-overlapping paths that emerged from a common initial trajectory before diverging into movement-specific structure, in all three areas. In the representative session shown, condition-dependent activity accounted for 57.9%/22.9%/39.2% of total variance in AIP/F5/M1, of which the first three PCs captured 60.3%/48.7%/67.1%. This pattern is illustrated from one representative session; trajectory separability and the variance-explained values were not tested or averaged across the six sessions (Figure 3).
• F5 required more independent activity patterns than AIP or M1 to describe movement-type differences, with mean D90/D95 values of 17.8/24.8 PCs in F5, versus 11.8/18.2 in AIP and 11.7/18.3 in M1. Direct session-level comparisons confirmed that this higher dimensionality in F5 was statistically significant relative to both AIP and M1, for D90 and D95, under both the historical and MAD-based reaction-time criteria (all *p* < 0.01, FWER-corrected) (Supplementary Figure S6; Supplementary methods 1.12).
• The degree to which combined-movement activity resembled a simple combination of its single-finger constituents varied by area. Alignment with constituent-defined activity was incomplete in AIP, F5, and M1 alike, and reconstruction from single-finger activity was generally incomplete as well, with numerically stronger reconstruction of F12 in M1 than in AIP or F5. These cross-area differences in alignment and reconstruction magnitude were not directly tested statistically between areas (Figure 4; Supplementary Figures S7–S9; Supplementary methods 1.13, 1.14).
• AIP showed an earlier numerical rise in condition-related tuning after cue onset, and the error-trial analysis showed that AIP and F5 activity preferentially matched the instructed condition after cue onset, whereas M1 did not; by the erroneous press, M1 matched the executed movement while AIP remained most instruction-matched and F5 stayed intermediate. The early AIP–F5 and AIP–M1 tuning-onset differences, and the error-trial cross-area ordering, were not directly tested as significant between areas; a significant AIP–M1 difference was confirmed later, near hold onset (Figure 2; Supplementary Figure S4; Supplementary methods 1.9, 1.15).

## 4 Discussion

In this work, we reported the simultaneously recorded neural activity on areas AIP, F5, and M1 in one macaque, and asked whether these three interconnected areas represent coordinated finger movements in the same way, both in the timing of task-related information and in the structure of that information. AIP showed the earliest numerical increase in condition-related tuning after the visual cue, although direct session-level comparisons did not establish a significant early AIP–F5 or AIP–M1 difference; a significant AIP–M1 difference emerged later around hold onset. The error-trial analysis showed complementary within-area patterns through the trial, with AIP remaining the most instruction-matched area, M1 shifting progressively toward the movement actually produced, and F5 falling consistently between the two. This relative cross-area ordering is interpreted descriptively because the statistical tests were conducted within each cortical area rather than directly between areas. At the population level, describing movement-type differences also required more independent activity patterns in F5 than in AIP or M1, a difference supported by direct session-level comparisons, and in all three areas combined-movement activity only partially resembled a simple combination of its single-finger constituents, with single-finger activity generally failing to reconstruct it. Thus, direct between-area evidence in this animal supports higher condition-dependent dimensionality in F5 and a later AIP–M1 tuning-prevalence difference, while the error-trial and compositional analyses provide additional descriptive evidence of area-specific organization.

Few intracortical studies have compared individual and combined finger movements, and none, to our knowledge, has simultaneously sampled AIP, F5, and M1 during this task (Baker et al., 2010; Ben Hamed et al., 2007; Nason et al., 2021; Shah et al., 2025; Arbuckle et al., 2022). The present recordings therefore provide a rare within-subject comparison across three nodes of the grasping network.

These area-specific patterns motivate hypotheses for BCI design rather than establish differences in decoder performance. Because overlap between combined- and single-finger activity was limited across AIP, F5, and M1, one possibility is that decoders may require some training directly from combined-movement data; such shared, low-dimensional structure has separately been shown to support BCI learning, behavioral readout, and cross-session stabilization (Sadtler et al., 2014; Gallego et al., 2017; Pandarinath et al., 2018; Degenhart et al., 2020). Descriptively, the degree of constituent structure varied across areas. M1 showed numerically stronger constituent-based reconstruction of F12 than AIP or F5, raising the hypothesis that generalization from single-finger training could differ across recording areas. However, this was not a direct comparison of decoder performance or calibration efficiency. We did not directly compare decoding performance across areas, dimensionalities, or calibration strategies, so these BCI implications should be regarded as hypotheses requiring explicit testing.

Accordingly, no single decoder architecture is implied by these data. Simple, linear approaches can support simultaneous finger control in some settings (Nason et al., 2021), while more flexible, non-linear approaches can improve naturalistic finger decoding in others (Temmar et al., 2024); whether the degree of model flexibility required depends on the recording area remains to be tested directly. Future work should test generalization from isolated digits across specific finger combinations and cortical areas, adding model flexibility where constituent-based decoding fails.

These area-specific differences extend to timing. AIP showed the earliest numerical increase in condition-related tuning after cue onset, although the early direct AIP–F5 and AIP–M1 comparisons did not reach significance across sessions, and remained preferentially matched to the instructed condition in error trials; F5 was intermediate, whereas M1 became more matched to the movement actually produced around erroneous presses. The relative cross-area ordering in the error-trial analysis is descriptive because the statistical tests assessed instructed-versus-executed correspondence within each area rather than direct differences between areas. These within-area patterns are consistent with AIP activity reflecting instructed/visuomotor information early and M1 activity reflecting execution-related information later, without establishing a causal serial transformation. Separately, F5 required more independent activity patterns than AIP or M1 to capture its condition-dependent activity. D90/D95 directly quantify the number of PCs required to explain a fixed proportion of condition-dependent variance and therefore support a higher-dimensional representation in F5, but do not by themselves distinguish a continuous from a categorical representational organization. Although previous work has proposed a broader continuum of hand configurations in F5 (Schaffelhofer and Scherberger, 2016), the present dimensionality analysis does not directly test that distinction.

Several limitations constrain interpretation. This is a single-subject case report (*N* = 1); the area-specific differences described are associations in this animal, not a general property of the primate grasping circuit, and require replication. Behavioral performance was also moderate, variable, and not fully optimized: the experiment ended for reasons unrelated to task performance, preventing further recording and behavioral optimization, so we cannot determine whether additional training would have improved or stabilized performance, and unequal task proficiency may have contributed to neural differences. Nevertheless, the simultaneous 192-channel recordings provide a rare within-subject comparison across AIP, F5, and M1 and establish the feasibility of comparing coordinated finger representations across this network.

Several limitations also concern our analytical assumptions and the lack of biomechanical controls. We subtracted the condition-averaged activity of each neuron for our population analyses (Supplementary methods); this removes shared “state-enabling” signals (timing, initiation), isolating coordination-related structure but discarding general timing dynamics that could themselves be useful for BCI gating. The absence of EMG, continuous force, and detailed kinematics also prevents us from assigning the non-compositional structure specifically to cortical computation rather than biomechanics, sensory feedback, or peripheral output (Schieber, 1991; Häger-Ross and Schieber, 2000; Arbuckle et al., 2022). Future studies combining large-scale neural recordings with simultaneous EMG, finger-force, and detailed kinematic measurements will help clarify how cortical populations coordinate multi-digit movements and relate to peripheral motor output (Kirsch et al., 2014; Schieber, 1995; Schieber and Santello, 2004; Schaffelhofer et al., 2015).

## Data Availability

The datasets presented in this article are not readily available because they are in use by other studies. Data is available upon reasonable request. Requests to access the datasets should be directed to Hansjörg Scherberger, hscherberger@dpz.eu.
