# Supplementary material for: Case Report: Representational geometry of multi-digit coordination in the macaque grasping network for intracortical BCIs
Source: Front Neurosci. 2026 Oct 27;20:1933898. doi: 10.3389/fnins.2026.1933898 (PMC13748289; doi:10.3389/fnins.2026.1933898)
Supplement: Supplementary file 1 [file Data_Sheet_1.pdf]

Supplementary Material

1 SUPPLEMENTARY METHODS

1.1 Basic procedures

One male rhesus macaque (*Macaca mulatta*, 10.5 kg) participated in this study. All animal care and experiments with the animals were conducted in accordance with the *Guidelines for the Care and Use of Mammals in Neuroscience and Behavioral Research* (National Research Council (US), 2003) and the *NC3Rs Guidelines* (NC3Rs, 2017), and were in agreement with German and European laws governing animal care. Authorization for conducting this study has been granted by the regional government office, the Animal Welfare Division of the Office for Consumer Protection and Food Safety of the State of Lower Saxony, Germany (permit no. 032/09 and permit no. 14-1442).

Previous to the array implantation, each animal received an MRI scan to locate anatomical landmarks for subsequent chronic implantation of microelectrode arrays. Each monkey was sedated (e.g., 10 mg/kg ketamine and 0.5 mg/kg xylazine, i.m.) and placed in the scanner (GE Healthcare 1.5T or Siemens Trio 3T) in a prone position. T1-weighted volumetric images of the brain and skull were obtained as described previously (Baumann et al., 2009). We measured the stereotaxic location of the arcuate and intraparietal sulci to guide placement of the electrode arrays.

Then in an initial procedure, a head post (titanium cylinder; diameter, 18 mm) was implanted on top of the skull (approximate stereotaxic position: midline, 40 mm anterior, 20° forward tilted) and secured with bone cement (Refobacin Plus; BioMed) and orthopedic bone screws (Synthes). After recovery from this procedure and subsequent training with head fixation, each animal was implanted in a second procedure with six floating microelectrode arrays (FMAs; MicroProbes for Life Science). Specifically, two FMAs were inserted in each area AIP, F5, and M1 (Fig. 2). FMAs consisted of 32 non-moveable monopolar platinum–iridium electrodes (impedance, 300–600 $k\Omega$ at 1 kHz), as well as two ground and two reference electrodes per array (impedance, $< 10\ k\Omega$). Electrode length ranged from 1.5 to 7.1 mm.

All surgical procedures were performed under sterile conditions and general anesthesia (e.g., induction with 10 mg/kg ketamine, i.m., and 0.05 mg/kg atropine, s.c., followed by intubation, 1–2% isoflurane, and analgesia with 0.01 mg/kg buprenorphene, s.c.). Heart and respiration rates, electrocardiogram, oxygen saturation, and body temperature were monitored continuously, and systemic antibiotics and analgesics were administered for several days after each surgery. To prevent brain swelling while the dura was open, the animal was hyperventilated mildly (end-tidal CO2, $\sim$30 mmHg), and mannitol was kept at hand. Animals were allowed to recover fully ($\sim$2 weeks) before behavioral training or recording experiments commenced.

After implantation, the monkey was habituated to sit in a primate chair with the head fixed. The left arm of the animal was restrained in a long tube to prevent interacting with the setup. The right arm went through a short tube with a manipulandum attached at the end, where he performed the finger task with his hand. The manipulandum detected finger flexion movements with five microswitches (Marquardt 1050, Rietheim-Weilheim, BW, Germany), arranged according to the fingertip positions of the monkey's right hand (Fig. 1). Thin metal layers were mounted on the index and middle finger microswitches to form an electrical circuit when the monkey correctly placed his fingers on the microswitches. This ensured the correct starting positions of the fingers and the hand.

Durign training and recordings, the right arm tube and the manipulandum were placed at a 45 degree angle upwards, in order to project the visual cues on the surface of the manipulandum. Cues were projected from a projector (Aiptek Q20, Taiwan, R.O.C) onto the white surface of the manipulandum in front of each finger to indicate the desired finger flexion (projection circles of ca. 1 cm in diameter). Eye movements were tracked with an infrared optical eye tracker (ISCAN, Woburn, MA, USA) and tracking was calibrated at the start of each session. All task relevant behavioral parameters (eye positions, stimulus presentation, switch activation) were controlled by custom-written behavioral control software implemented in LabVIEW (National Instruments, Austin, TX, USA).

1.2 Task paradigm

The monkey was trained to perform an individual and combined finger flexion task with five different conditions, including individual thumb (F1), index (F2), and middle finger (F3), and the combined finger movements of F12 and F23 (Fig. 1a). A trial was initiated when the monkey placed his index and middle finger on the microswitches of the manipulandum. When the electrical circuit formed, the three initial yellow dots disappeared. A fixation red dot appeared between the index and middle finger cues and the monkey was required to fixate during the entire trial. After fixating for 500 ms (fixation epoch), the cue appeared as green dots in front of the instructed finger(s) for 500 ms (cue epoch). Combined finger movements were indicated with two dots. Cues were presented in a pseudorandom order, where successful trials were removed and the unsuccessful trial stayed in the pool. The monkey was allowed to press after the cue disappeared (go epoch, maximum 1000 ms). After pressing the correct microswitch(es) for 100 ms (hold epoch), a drop of juice was delivered as a reward. When the microswitches were pressed, blue dots appeared at the same positions of the cues as visual feedback. This helped the monkey identify both a correct press and an incorrect press. Incorrect trials were aborted immediately.

1.3 Behavioral data analysis and trial inclusion

Behavioral success rate was calculated separately for each movement condition and recording session. Only trials that reached the cue epoch were included in the success-rate denominator because movement information was first available at cue presentation.

Since the movement range of the microswitches was very small (a few millimeters), the time from go-cue onset to microswitch press was almost entirely reaction time. We refer to the original reaction-time-exclusion approach as the historical analysis throughout, since it uses the fixed thresholds from our initial analysis and is retained here for comparison with the newer, MAD-based sensitivity analysis described below. The historical analysis excluded long-reaction-time trials using fixed thresholds chosen from the right-skewed reaction-time distributions (median ≈ 0.1 s for single-finger movements and ≈ 0.3 s for combined movements; Supplementary Fig. S2): 0.20 s for single-finger movements (F1, F2, F3) and 0.42 s for combined movements (F12, F23).

For coordinated movements, both appropriate microswitches had to be activated within 100 ms to distinguish simultaneous from sequential responses. Across the six sessions, 1,924 trials were originally task-successful; 1,538 trials remained after applying this coordinated double-movement validity criterion. This 1,538-trial pool therefore served as the behaviorally valid pre-reaction-time (pre-RT) dataset.

These historical thresholds retained 1,272 of the 1,538 behaviorally valid pre-RT trials (82.7%). Relative to the 1,924 originally successful trials, the final historical analysis set therefore represented 66.1% retention.

As a sensitivity analysis, reaction-time thresholds were also defined independently for each movement condition using the median plus three scaled median absolute deviations (scaled MAD $= 1.4826 \times$ median|RT $-$ median(RT)|). The resulting thresholds were 0.322 s (F1), 0.506 s (F2), 0.597 s (F12), 0.287 s (F3), and 0.460 s (F23), retaining 1,463 of 1,538 pre-RT trials (95.1%; Supplementary Fig. S2). For analyses repeated under the MAD criterion, condition-dependent population activity, PCA eigenspectra, and downstream dimensionality-dependent analyses were recomputed from the MAD-selected trials rather than inheriting quantities estimated from the historical dataset.

1.4 Chance level models and behavioral statistical significance

To evaluate whether the monkey's behavioral performance was guided by the visual cues rather than random guessing, we established two distinct chance level models. Under the nominal class-selection model, we assumed that the monkey had learned the task rules (i.e., that a trial required executing one of the five cued configurations: F_1, F_2, F_3, F_{12}, or F_{23}) and randomly chose among these cued classes, resulting in a nominal chance probability of 20% (1/5). Under the combinatorial press model, we assumed the monkey did not restrict its choices to the cued movement set but instead randomly activated any combination of the three active finger switches (Thumb, Index, Middle) on the manipulandum. Since there are $2^3 - 1 = 7$ possible non-empty combinations, the combinatorial chance probability of executing the correct combination is $\approx 14.3\%$ (1/7). To test the statistical significance of the behavioral success rates, we performed a one-sided binomial test separately for each movement condition. Trials were pooled across the six recording sessions (Rec4–Rec9) for this analysis; the tests were not performed separately for each session and were not based on the across-session mean success rate. For each condition, k was the total number of successfully completed trials across the six sessions and n was the total number of trials for that condition that reached cue presentation. The same k and n were tested against the two null probabilities, $p_0 = 0.20$ and $p_0 = 1/7 \approx 0.143$.

The exact pooled counts and one-sided binomial p-values were:

Condition	Correct trials k	Cued trials n	Success rate	p vs. 20% chance	p vs. 14.3% chance
F1	425	792	53.66%	2.65×10^{-97}	1.89×10^{-148}
F2	379	970	39.07%	2.02×10^{-42}	3.53×10^{-80}
F3	477	758	62.93%	9.02×10^{-146}	4.44×10^{-207}
F12	312	976	31.97%	8.78×10^{-19}	1.29×10^{-44}
F23	331	555	59.64%	1.30×10^{-92}	2.69×10^{-134}

Across conditions, this corresponded to 1,924 successful trials among 4,051 trials that reached cue presentation.

Session-specific success rates are shown in Supplementary Fig. S1 to illustrate behavioral variability across recordings but were not the observational units used in the binomial chance tests.

1.5 Behavioral result details

Across the six recording sessions, the monkey successfully completed 1,924 of 4,051 trials that reached cue presentation (47.5%; approximately 48%), corresponding to approximately 321 successful trials per session. Pooled condition-specific success rates across the six sessions were 54% for F1, 39% for F2, 63% for F3, 32% for F12, and 60% for F23. The marked difference between the two combined movements

indicates that generic difficulty in coordinating two digits is insufficient to explain performance. Session-by-session analysis showed that F12 remained comparatively difficult across recordings, whereas F23 performance was relatively high in most sessions except Rec5; overall performance fluctuated without a clear monotonic trend (Supplementary Fig. S1). Possible contributors include digit-specific biomechanical coupling, movement strategy, manipulandum or switch demands, and condition-specific task proficiency. Because continuous kinematic, force, and EMG measurements were not available, these possibilities cannot be distinguished.

Recording began after the animal had acquired the task sufficiently for the planned neural-recording and real-time-decoding experiments, although performance was not fully optimized and remained variable across movement conditions. After six recording sessions, the implanted arrays had to be explanted for reasons unrelated to the finger-movement experiment, preventing further recording and additional behavioral optimization.

Under the historical reaction-time thresholds, 1,272 trials remained for the principal neural analyses (212 trials per session on average). Historical mean $\pm$ SD reaction times were 81 ± 51 ms for F1, 97 ± 58 ms for F2, 83 ± 51 ms for F3, 221 ± 83 ms for F12, and 203 ± 80 ms for F23. Each movement condition retained, on average, more than 13 correct trials per session.

1.6 Neural recordings and spike sorting

Neural signals recorded simultaneously from a total of 192 channels of six arrays (in areas AIP, F5, and M1) were amplified and digitally stored using a 256-channel TDT RZ2 (sampling rate 24.414 kS/s; 0.6–10000 Hz band-pass hardware filter, Tucker-Davis Technologies, Alachua, FL, USA). Preprocessing before spike detection includes filtering and denoising: raw neural signals were first low-pass filtered with a median filter (window length 3 ms). The filtered signal was then subtracted from the original data, and this corresponds to a nonlinear high-pass filter. A non-causal Butterworth low-pass filter (5000 Hz; 4th order) was then applied to the subtracted signal. Principal component analysis (PCA) was applied for all electrodes of each array to eliminate common noise sources, as described previously (Musial et al., 2002). To ensure that no individual electrodes were discarded, PCs with a normalized coefficient larger than a conservatively chosen 0.36 threshold were retained (Dann et al., 2016).

Spike waveforms were detected and semi-automatically sorted using a modified version of the offline spike sorter Wave_clus (Quiroga et al., 2004; Michaels et al., 2015; Dann et al., 2016). Spike detection was performed by amplitude thresholding at $4\sigma_n$, where σ_n is an estimate of the standard deviation of the background noise. Feature extraction of spike shapes was conducted by testing outcome from PCA, wavelet transform (WT), and original waveforms with the Lilliefors test separately. The WT is a time-frequency representation of the signal, providing an optimal resolution in both time and frequency domains. The Wavelet coefficients characterize the spike shapes at different scales and times. The Lilliefors test compares the cumulative distribution function of the data with that of a Gaussian distribution with the same mean and variance. Clustering of the spikes by the selected features was done by superparamagnetic clustering, an idea based on simulated interactions between each data point and its K-nearest neighbors. In Wave_clus, a graphical user interface (GUI) is implemented in Matlab to manually select the clusters and unassigned waveforms are matched to the templates of the desired clusters by a linear discriminant analysis (LDA) classifier. After the waveforms were semi-automatically sorted, the different averaged templates were redetected to discover the overlaid waveforms (Gozani and Miller, 1994). The redetection was achieved by convolution of the filtered signals with the waveform templates and classified by an LDA classifier. After each redetection, the current template was subtracted from the filtered signal to reduce artifacts during the

next redetection. With this procedure, spikes with a temporal overlap up to 0.2 ms could be redetected. If the firing rate changed more than 30% between the first 10 min and the last 10 min of the recording session, the corresponding unit was excluded from all further analyses.

After spike sorting, we classified units into single- or non-single unit according to five criteria (Dann et al., 2016): (1) the absence of short (1.2 ms) intervals in the inter-spike interval histogram for single units; (2) the homogeneity and spike density of the detected waveforms; (3) the separation of waveform clusters in the projection of the first 17 features (a combination for optimal discriminability of PCs, single values of the wavelet decomposition, and samples of spike waveforms) detected by Wave_clus; (4) the presence of well-known waveform shapes characteristics for single units; and (5) the shape of the inter-spike interval distribution.

1.7 Neural recording result details

The analyzed dataset included six sessions recorded simultaneously in AIP, F5, and M1 using six 32-channel floating microelectrode arrays (two in each area). There were on average 63 ± 6.4 single and multi-units (mean $\pm$ SD) in AIP (16.7 ± 6.6 single units), 72.5 ± 6.0 units in F5 (24.7 ± 4.4 single units), and 92.2 ± 10.1 units in M1 (38.0 ± 5.1 single units). Units were classified as task-related if they showed significant firing-rate differences between movement conditions at any time during the trial using a cluster-based permutation test. In AIP, F5, and M1, 74%, 78%, and 79% of units were task-related, respectively.

Consistent with previous finger-movement studies in M1 (Schieber and Hibbard, 1993; Poliakov and Schieber, 1999), many units in AIP, F5, and M1 were tuned to multiple movement conditions and showed heterogeneous temporal profiles (Supplementary Fig. S3). To summarize tuning dynamics, units in each area were ordered by significant tuning onset. During the first 250 ms after cue onset, the mean percentage of tuned units was 12.3% in AIP, 8.1% in F5, and 7.5% in M1. Later in the trial, tuning became more prevalent in M1, reaching 69.4% shortly before the hold epoch, compared with maxima of 62.0% in F5 and 59.9% in AIP. An initial pooled unit-level analysis identified significant cortical-area effects during an early cue-period cluster (approximately 60–220 ms after cue onset, cluster-corrected $p = 0.007$) and a later cluster spanning approximately 210 ms before to 60 ms after hold onset ($p = 0.004$). However, the direct session-level between-area analysis (direct AIP–F5 and AIP–M1 comparisons) using the six recording sessions as the resampling unit did not identify a significant between-area difference during the early cue period (AIP–F5: minimum cluster-corrected $p = 0.283$; AIP–M1: $p = 0.070$). A significant session-level between-area difference was detected later between AIP and M1, spanning approximately 100 ms before to 300 ms after hold onset, during which the proportion of tuned units was lower in AIP than in M1 (cluster-corrected $p = 0.009$). No significant AIP–F5 cluster was detected. Thus, the early AIP increase is treated as a descriptive feature of the sampled unit population rather than as evidence for a statistically established earlier tuning onset across sessions.

These correct-trial timing data therefore suggest an earlier emergence of task-condition information in AIP at the level of the observed tuning profile, but they cannot distinguish visual/instruction-related activity from motor intention because cue identity and instructed movement are coupled on correct trials. We therefore performed an additional error-trial analysis that directly compared instructed-condition and executed-condition population correspondence (Section 1.15; Supplementary Fig. S4).

1.8 Peristimulus time histograms (PSTHs)

After sorting, spikes were binned into non-overlapping 1 ms windows and smoothed with a Gaussian kernel ($\sigma = 60$ ms). Data were aligned to cue onset and hold onset. We chose the cut-off time to be 250 ms after the cue onset and 350 ms before the hold epoch, so that there were small overlaps and smooth transitions. PSTHs were then calculated by averaging over all trials per condition and unit.

1.9 Cluster-based permutation test (CBPT)

To test for significant differences in firing rate between conditions and to adequately deal with the multiple comparison problem of multiple time points, we used a cluster-based permutation test (Maris and Oostenveld, 2007). For every time point, the conditions were tested with a 1-way ANOVA. All the time points with F-values larger than some threshold (this threshold only affects the sensitivity of the test, not the false alarm rate) were selected, clustered on the basis of temporal adjacency, and F-values summed within each cluster. The condition labels of trials were randomly rearranged to create 1000 shuffled data sets, and the same procedure was applied to each shuffled data set. In each shuffled data set, only the cluster with the highest summed F-value was selected to create a null distribution of F-values. The permutation p-value is the proportion of shuffled data sets with a cluster-level F-value higher than the original data. The critical alpha-level for the p-value was set at 0.05. When dealing with multiple comparisons, clustering adjacent time samples is less conservative than Bonferroni correction.

The test described above was used to determine movement-condition tuning within individual recorded units. To compare the temporal prevalence of tuning across cortical areas, we performed two analyses with different observational levels: an initial pooled unit-level analysis and a direct session-level analysis. In the initial pooled unit-level analysis, the binary tuning-status time series of recorded units were pooled across the six recording sessions within each cortical area. At each time point, tuning status was compared among AIP, F5, and M1 using a one-way ANOVA. Whole unit time series were randomly permuted across cortical-area labels 1,000 times. Temporally adjacent samples exceeding an uncorrected threshold of $p < 0.05$ were grouped into clusters, and cluster mass was defined as the sum of F-values within each cluster. The maximum cluster mass from each permutation was used to construct the null distribution. Because this analysis treats pooled recorded units as the observational level rather than preserving their nesting within recording sessions, it serves as an initial characterization of the sampled unit population rather than as the primary test of between-session consistency.

In the direct session-level analysis, conducted to evaluate between-area differences while preserving the repeated-session structure, we calculated the proportion of tuned units separately for AIP, F5, and M1 in each of the six simultaneous recording sessions at every time point. The planned AIP–F5 and AIP–M1 contrasts were tested using paired t-statistics across sessions at each time point. Temporally adjacent samples exceeding a two-sided cluster-forming threshold of $p < 0.05$ were grouped into clusters, with cluster mass defined as the sum of the absolute t-statistics within each cluster. Cortical-area labels were permuted within each recording session, thereby preserving the pairing of simultaneously recorded areas. Because each of the six sessions contained all three cortical areas, all $3!^6 = 46{,}656$ possible within-session area-label permutations were enumerated. For each permutation, the largest cluster mass across both planned contrasts and all time points was retained to form a common null distribution, thereby controlling the family-wise error rate across time and the two pairwise comparisons. Cluster-level p-values were calculated as the proportion of permutations in which the maximum cluster mass equaled or exceeded the observed cluster mass, and clusters with corrected $p < 0.05$ were considered significant. Recording session ($n = 6$) was therefore the resampling unit for these direct between-area comparisons. Because

all six sessions were obtained from the same animal, these tests assess within-animal consistency across recording sessions rather than population-level differences across animals.

1.10 Mean subtracted neural activity

In order to remove the "condition-invariant" part and preserve the "condition-dependent" part of each unit, the mean firing rate of all five conditions at each time point was subtracted from each condition (Rouse and Schieber, 2018). This was performed for both trial averaged and single trial neural activity (only for multiple linear regression in this study). The mean firing rate of all five conditions was obtained by first averaging across all trials in each condition and then averaging across conditions. This ensures that the mean firing rate is not biased by conditions with higher trial numbers.

1.11 Cluster analysis and measure of correlation between condition pairs

For the purpose of cluster analysis, a single numeric measure is required for each movement. For this, we used the mean subtracted firing rate averaged over a time window of 200 ms (100 ms before hold epoch till end of the hold epoch) to capture the movement-related firing rate changes, and only neurons tuned during this time window were included in the analysis. Each of the mean subtracted firing rates was then normalized to a unit vector by dividing by the sum of the root mean squares of all conditions (five in total). For each neuron, the normalized firing rates of the five finger movements were considered as one data point in a five-dimensional space for hierarchical cluster analysis based on Euclidean distance (Matlab function: `linkage`). The output was displayed as a dendrogram (Matlab function: `dendrogram`) with a colormap showing the rearranged order of units according to it.

To test whether the tuning of one movement was positively correlated with another in the neuronal population, we calculated the Spearman's rank correlation coefficient between all condition pairs (five conditions resulted in ten condition pairs) using the same input as for the cluster analysis. We built a matrix containing the averaged mean subtracted firing rates of the five conditions. This matrix had c rows by n columns, where c is the number of conditions and n is the number of units in one area. For condition pair ij (both i and j are rows in the data matrix), units in i and j rows were shuffled differently 1000 times to generate chance level correlation coefficient (null distribution). Correlation coefficient values were considered significant if they exceeded the 95%-percentile in the null distribution. The ten correlation coefficient values for all condition pairs were calculated for the three areas separately.

1.12 Principal component analysis (PCA) and objective dimensionality

Principal component analysis (PCA) was applied to square-root-transformed, trial-averaged condition-dependent PSTHs (Matlab functions: `princomp`, `svd`) (Yu et al., 2009). The square-root transform reduced the influence of neurons with very strong responses while preserving lower-rate responses. A $c(t) \times n$ data matrix was formed, where n was the number of recorded neurons and $c(t)$ represented the selected movement conditions over time. PCA identified ordered orthogonal axes capturing decreasing amounts of variance in the condition-dependent population activity.

Condition-dependent activity was obtained by subtracting, for each neuron and time point, the mean response across the five movement conditions. In the representative session shown in main-text Fig. 3, this condition-dependent component, expressed as a fraction of the total trial-averaged population variance, accounted for 57.9% in AIP, 22.9% in F5, and 39.2% in M1 (obtained using demixed principal component analysis, dPCA). These percentages therefore do not represent variance explained by the first three PCs.

The first three PCs were used in main-text Fig. 3 to visualize the dominant condition-dependent population trajectories. In the same representative session, the first three PCs captured 60.28% of the condition-dependent variance in AIP, 48.71% in F5, and 67.11% in M1. Thus, the three-dimensional trajectories visualize a substantial portion of the movement-related structure but do not provide a complete representation of the population geometry.

To quantify dimensionality without choosing a fixed number of PCs, for each recording session and cortical area independently we defined D90 and D95 as the minimum numbers of PCs required to explain 90% and 95% of the condition-dependent variance, respectively. Under the historical reaction-time (RT) criterion, mean D90/D95 values across sessions were 11.8/18.2 PCs in AIP, 17.8/24.8 PCs in F5, and 11.7/18.3 PCs in M1 (Supplementary Fig. S6). The same procedure was repeated after MAD-based trial selection, with the PCA eigenspectrum and D90/D95 values recalculated independently from the MAD-selected data.

Direct between-area comparisons were performed separately for the historical and MAD-based RT criteria using recording session ($n = 6$) as the resampling unit. The four planned contrasts were F5–AIP and F5–M1 for D90 and D95. Cortical-area labels were permuted within each recording session, and all $3!^6 = 46{,}656$ possible within-session area-label permutations were enumerated. For each permutation, the largest absolute paired t-statistic across the four planned comparisons was retained to construct a common maximum-statistic null distribution, thereby controlling the family-wise error rate across the four comparisons.

Under the historical RT criterion, dimensionality was significantly higher in F5 than in both AIP and M1 for D90 (F5–AIP: $p = 0.0045$; F5–M1: $p = 0.0027$) and D95 (F5–AIP: $p = 0.0051$; F5–M1: $p = 0.0021$; all six values FWER-corrected). The same pattern was retained after MAD-based trial selection (D90: F5–AIP, $p = 0.0087$; F5–M1, $p = 0.0020$; D95: F5–AIP, $p = 0.0071$; F5–M1, $p = 0.0023$).

1.13 Variance alignment analysis and dimensionality sensitivity

We applied a modified variance-alignment analysis (Raposo et al., 2014) to quantify how strongly a combined movement occupied a state space defined by its two constituent single-finger movements. For F12, the reference state space was defined by F1 and F2 and the unrelated control was F3; for F23, the reference was defined by F2 and F3 and the unrelated control was F1.

In the initial analysis, square-root-transformed condition-dependent activity was first denoised by retaining eight PCs. These eight PCs explained $84.8 \pm 3.5\%$, $73.0 \pm 3.2\%$, and $85.2 \pm 1.6\%$ (mean $\pm$ SD across recordings) of condition-dependent variance in AIP, F5, and M1, respectively. PCA was then performed on the two related single-finger movements to define the constituent reference space. The combined movement and control movements were rotated into this space, and cumulative variance alignment was quantified across dimensions 1–8. Alignment values were normalized relative to the maximal variance that could be captured in the same number of dimensions. A summary alignment index was defined as the normalized area between the observed alignment curve and the chance curve, ranging from -1 (maximally misaligned) to 1 (perfectly aligned). Chance was estimated from 10,000 random orthogonal orientations in the eight-dimensional space.

To test sensitivity to the initially retained dimensionality, the complete analysis was repeated after retaining 3, 5, 8, 10, 12, 15, or 20 PCs. For each setting, constituent-specific alignment was summarized as Δ alignment index = alignment(DM) $-$ alignment(nrSM), where DM denotes the corresponding double movement and nrSM the unrelated single-movement control. Positive values therefore indicate preferential

alignment of the combined movement with its constituent-defined state space, whereas negative values indicate greater alignment of the unrelated control (Supplementary Fig. S7, panel A).

We additionally repeated the analysis using objective dimensionalities determined separately for each session and cortical area: D90 or D95, defined as the numbers of PCs required to retain 90% or 95% of condition-dependent variance. This was performed first with the historical reaction-time-selected data (Supplementary Fig. S7, panel B) and then after MAD-based trial selection, with PCA and D90/D95 recalculated independently from the MAD-selected data (panel C). Summary points show mean $\pm$ SEM across sessions. Recording sessions ($n = 6$) were treated as the observational units for the session-level summaries. No direct statistical comparison of alignment magnitude among AIP, F5, and M1 was performed; cross-area differences in alignment are therefore interpreted descriptively.

1.14 Multiple linear regression and dimensionality sensitivity

Multiple linear regression (Matlab function: `regress`) was used to test whether the linear combination of constituent single-finger trajectories reconstructed the corresponding combined-movement trajectory better than an unrelated single-finger control. For F12, F1 and F2 were predictors and F3 was the unrelated control; for F23, F2 and F3 were predictors and F1 was the unrelated control. The reconstruction model can be written as:

$$X_{12} = X_1\beta_1 + X_2\beta_2 + \beta_0 \tag{S1}$$

where X_{12} is the combined-movement trajectory, X_1 and X_2 are the constituent trajectories, β_1 and β_2 are regression coefficients, and β_0 is the constant term.

The initial analysis projected square-root-transformed condition-dependent PSTHs onto the first five PCs. These five PCs explained $77.2 \pm 4.9\%$, $61.4 \pm 3.7\%$, and $77.3 \pm 2.2\%$ (mean $\pm$ SD across recordings) of condition-dependent variance in AIP, F5, and M1, respectively. To reduce overfitting, combined-movement trials were repeatedly divided into equally sized training and test sets; all possible splits were used when computationally feasible, otherwise 5,000 random splits were sampled. Regression coefficients were estimated on the training data and evaluated on held-out test trajectories. The unrelated control was reconstructed using the same procedure and matched trial counts. The original fixed-five-PC group summary is shown in Supplementary Fig. S8.

For the dimensionality robustness analysis, reconstruction was repeated after retaining 3, 5, 8, 10, 12, 15, or 20 PCs and summarized as $\Delta R^2 = R^2_{\text{DM}} - R^2_{\text{nrSM}}$. Positive ΔR^2 indicates that the constituent trajectories reconstructed the corresponding combined movement better than the unrelated control; negative values indicate poorer reconstruction of the combined movement (Supplementary Fig. S9, panel A).

The regression was also repeated at session- and area-specific D90 and D95 dimensionalities using the historical reaction-time-selected data (Supplementary Fig. S9, panel B) and after applying the MAD-based reaction-time criterion (panel C). For the MAD analysis, PCA and D90/D95 were recalculated independently from the MAD-selected data. Recording sessions ($n = 6$) were the independent observational units, and summary points show mean $\pm$ SEM across sessions. Across dimensionality choices, the predominant pattern was limited constituent-specific reconstruction, with numerically stronger reconstruction of F12 in M1 than in AIP or F5. The same descriptive pattern was maintained under the MAD-based criterion. No direct statistical comparison of reconstruction magnitude among cortical areas was performed; cross-area differences in reconstruction are therefore interpreted descriptively.

1.15 Error-trial analysis of instructed- and executed-condition correspondence

Correctly performed trials were used to construct condition-specific population templates. Error trials were selected when the instructed movement and the subsequently executed microswitch response differed. For each error type and time point, the error-trial population vector was compared with the correct-trial template for the instructed condition and with the template for the actually executed condition using partial correlation. The instructed-template correlation controlled for the executed template, and the executed-template correlation controlled for the instructed template.

Correlation coefficients were Fisher z-transformed and relative correspondence was defined as $\Delta z = z(r_{\text{executed}}) - z(r_{\text{instructed}})$. Negative values therefore indicate preferential correspondence with the instructed-condition representation, whereas positive values indicate preferential correspondence with the executed-condition representation. Error types containing at least three trials within a recording session were retained. Eligible error types were averaged within each recording session before group-level inference, and the six recording sessions were treated as the independent observational units.

The analysis was performed after alignment to cue onset, cue offset/go, and the executed erroneous microswitch press. Temporal significance was assessed using exact two-sided session-level sign-flip cluster tests ($p < 0.05$); curves and shaded regions in Supplementary Fig. S4 show the across-session mean $\pm$ SEM. These statistical tests assess instructed- versus executed-condition correspondence within each cortical area. They do not directly test differences in Δz among AIP, F5, and M1; consequently, the relative ordering of the three areas is interpreted descriptively. Cue-aligned analysis showed an instructed-condition preference in AIP and F5 after cue onset, whereas M1 showed no comparable early preference. Go-aligned activity subsequently diverged across areas, with AIP remaining more instruction-matched, F5 approaching intermediate correspondence, and M1 shifting toward the actually executed condition. Around the erroneous microswitch press, M1 preferentially corresponded to the movement that was actually produced, while AIP remained more instruction-matched and F5 showed an intermediate pattern.

Because AIP showed a visible pre-cue bias in the cue-aligned trajectory, a baseline-control analysis was performed by subtracting the mean pre-cue Δz from -500 to -50 ms separately within each session. A post-cue shift toward the instructed-condition representation remained after baseline subtraction. Across sessions, the paired change from the pre-cue baseline to 0–250 ms after cue onset was -0.229 in Δz, with all six sessions shifting in the same direction (exact two-sided sign-flip $p = 0.0312$; Supplementary Fig. S4, panel E). Because the visual cue remains coupled to the instructed condition, this analysis distinguishes instructed-condition from executed-condition correspondence rather than providing a pure visual-versus-motor dissociation.

2 SUPPLEMENTARY FIGURES AND TABLES

2.1 Supplementary figures

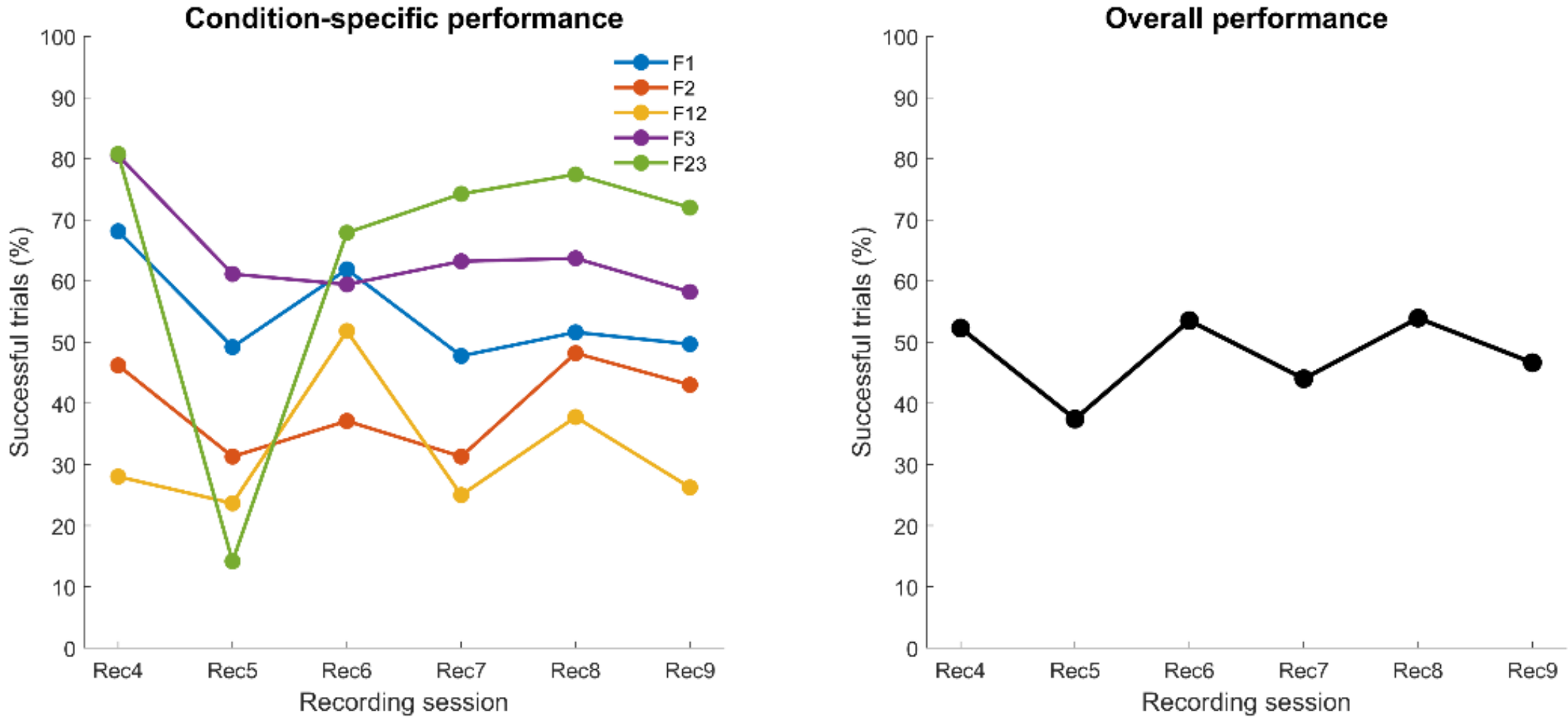

Figure S1. Behavioral performance across the six recording sessions. **(A)** Condition-specific success rates for the five instructed movement conditions across Rec4–Rec9. F1, F2, and F3 are single-finger movements; F12 and F23 are simultaneous thumb-index and index-middle movements. F12 remained comparatively difficult across recordings, whereas F23 performance was relatively high in most sessions except Rec5. **(B)** Overall success rate across all five movement conditions for each session. Overall performance fluctuated between sessions rather than showing a clear monotonic trend across the six recordings, indicating substantial session-to-session behavioral variability.

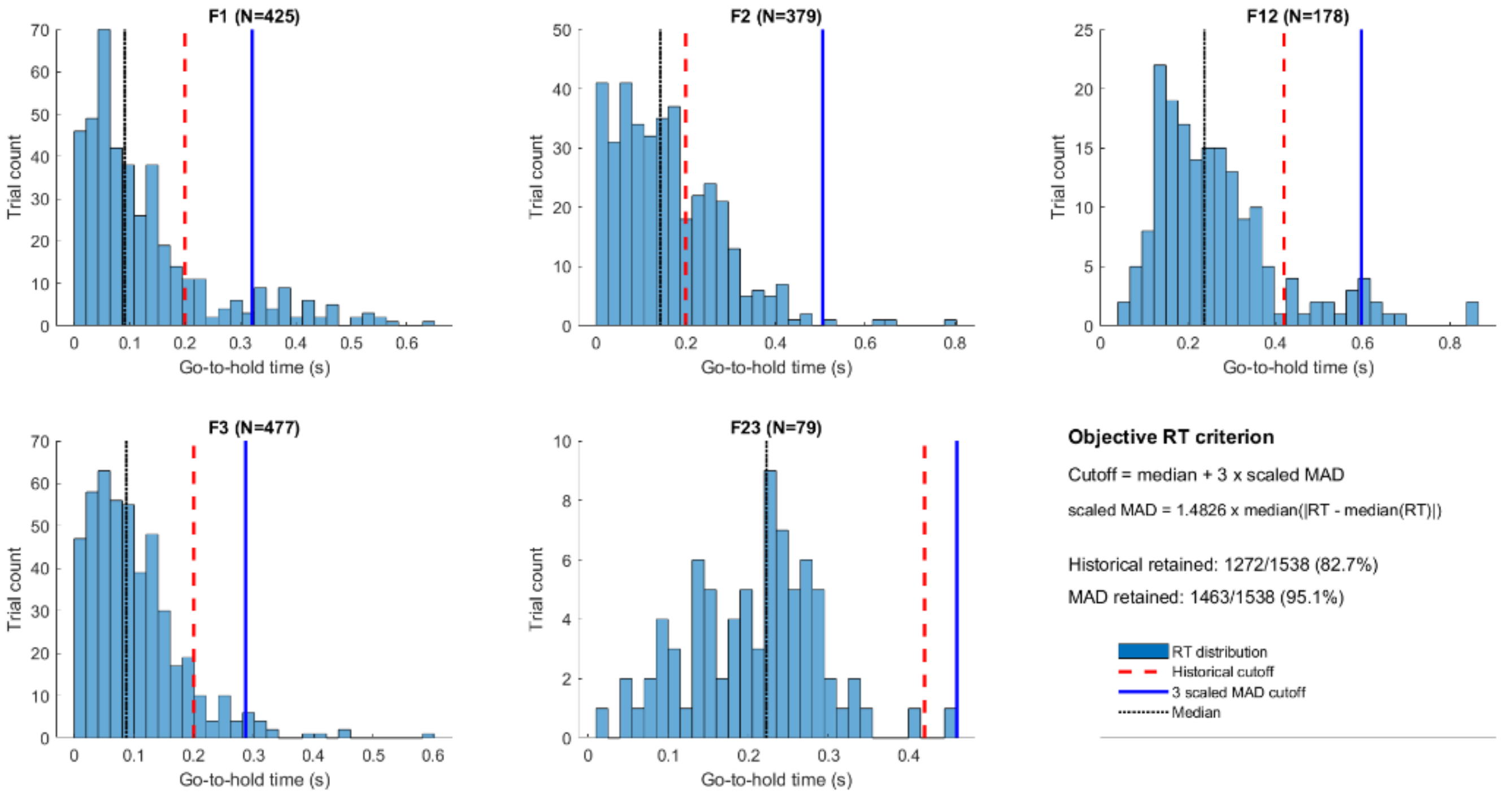

Figure S2. Sensitivity of reaction-time exclusion to the thresholding criterion. Reaction-time distributions for the five movement conditions were reconstructed from all behaviorally valid trials across the six recording sessions prior to reaction-time exclusion. Dashed red lines indicate the historical thresholds used in the original analysis (0.20 s for F1, F2, and F3; 0.42 s for F12 and F23). Solid blue lines indicate condition-specific robust thresholds defined as the median plus three scaled median absolute deviations (scaled MAD $= 1.4826 \times \text{median}|\text{RT} - \text{median}(\text{RT})|$); dotted black lines indicate the median of each distribution. The resulting MAD-based thresholds were 0.322, 0.506, 0.597, 0.287, and 0.460 s for F1, F2, F12, F3, and F23, respectively. Across all conditions, the historical criterion retained 1,272 of 1,538 behaviorally valid trials (82.7%), whereas the MAD-based criterion retained 1,463 trials (95.1%).

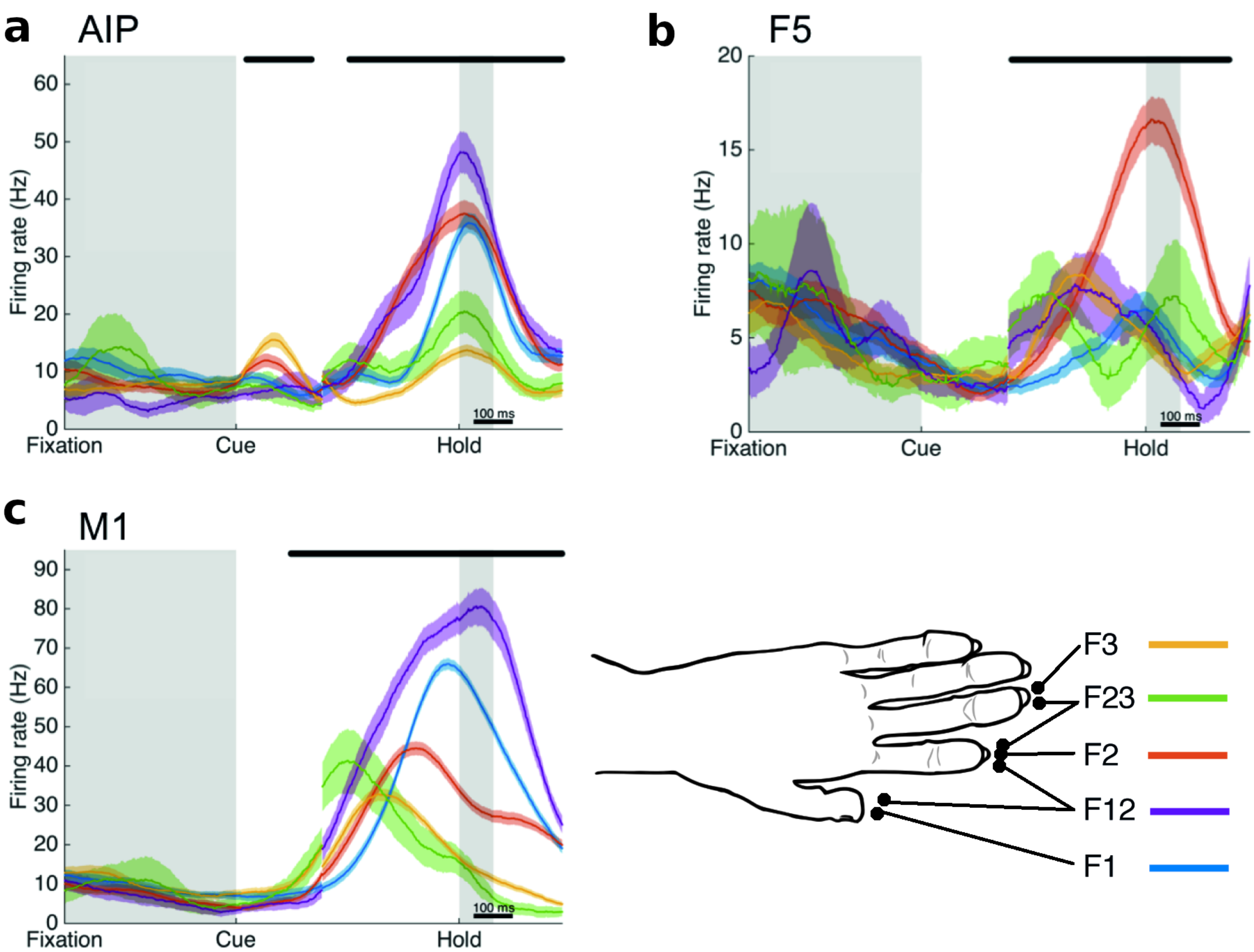

Figure S3. Example units from areas AIP, F5, and M1. **(a–c)** Peristimulus time histograms (PSTHs) of example units from area AIP (**a**; multi-unit), F5 (**b**; single unit), and M1 (**c**; single unit) across the five finger-movement conditions. Onsets of the fixation, cue, and hold epochs are marked; trials are aligned to cue onset in the left segments and hold onset in the right segments. Shaded regions indicate standard error. Time intervals with significant movement tuning are indicated by horizontal bars ($p < 0.05$, cluster-based permutation test).

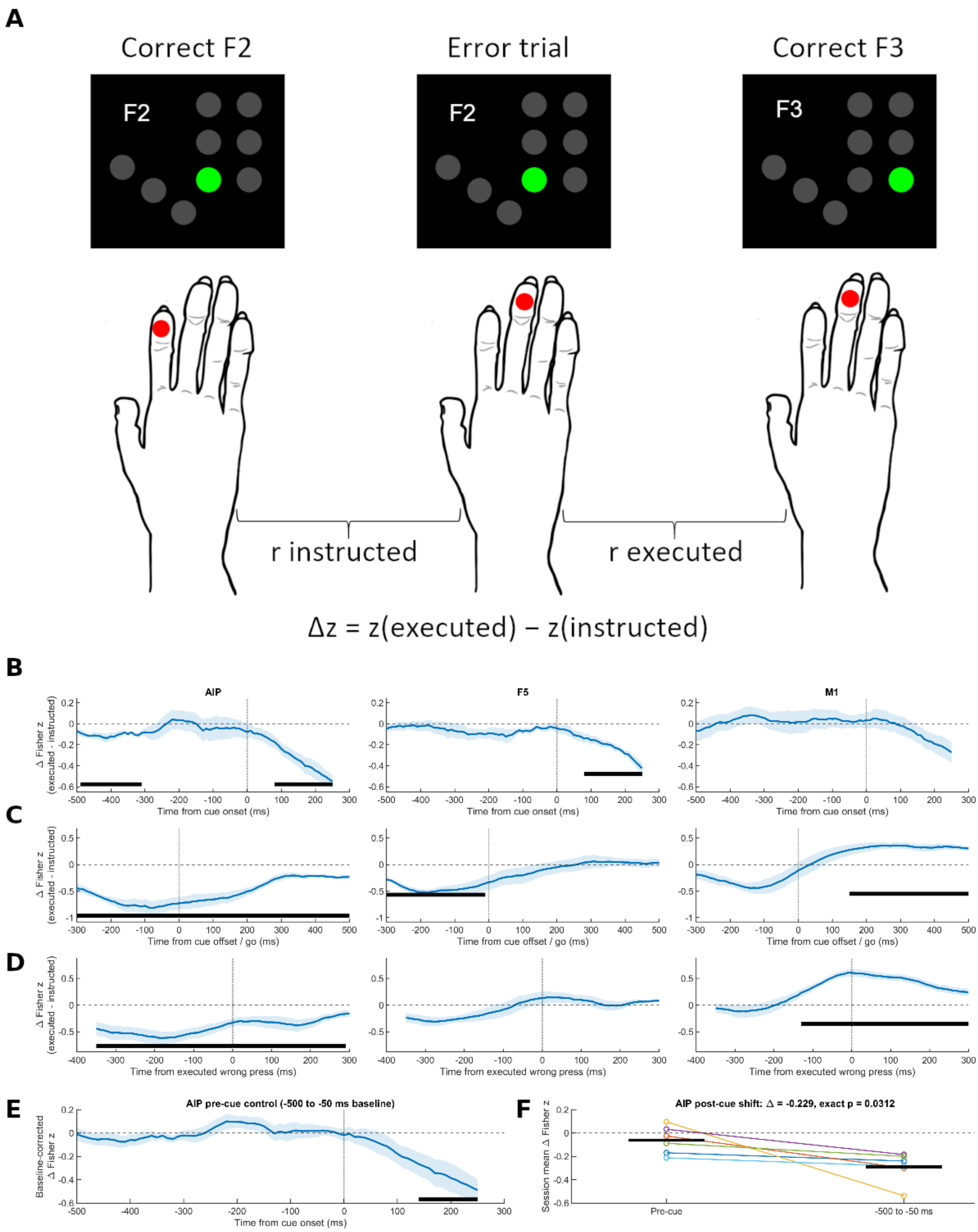

Figure S4. Error trials dissociate instructed-condition and executed-condition population representations across the frontoparietal grasping network. **(A)** Schematic of the analysis. Correct trials defined templates for each movement condition. For an error trial, the population pattern was compared with templates for the instructed and actually executed conditions using partial correlation.

Figure S4. (Continued) Correlations were Fisher z-transformed and relative correspondence was defined as $\Delta z = z(r_{\text{executed}}) - z(r_{\text{instructed}})$; negative values indicate preferential instructed-condition correspondence and positive values indicate preferential executed-condition correspondence. **(B–E)** Temporal evolution of Δz after averaging eligible error types within each session; error types required at least three trials and sessions ($n = 6$) were the independent units. Curves show mean $\pm$ SEM and black horizontal bars indicate significant temporal clusters from exact two-sided session-level sign-flip cluster tests ($p < 0.05$). Negative values: instructed-condition matched. Positive values: executed-condition matched. **(B)** Cue-onset alignment: AIP and F5 became preferentially instructed-condition matched after cue presentation, whereas M1 showed no comparable early preference. **(C)** Cue-offset/go alignment: AIP remained instruction-matched, F5 approached intermediate correspondence, and M1 shifted toward the executed condition after go. **(D)** Erroneous-press alignment: M1 preferentially corresponded to the movement actually produced, while AIP remained more instruction-matched and F5 was intermediate. The significance bars test instructed- versus executed-condition correspondence within each cortical area; differences in the relative position of the AIP, F5, and M1 curves are descriptive and were not subjected to direct between-area statistical tests. **(E)** AIP pre-cue control: after subtracting the -500 to -50 ms baseline within each session, a post-cue instructed-condition shift remained. **(F)** Same as (E) but for individial sessions at the pre-cue and post-cue time points. All six sessions shifted in the same direction (mean paired Δz change = -0.229, exact two-sided sign-flip $p = 0.0312$).

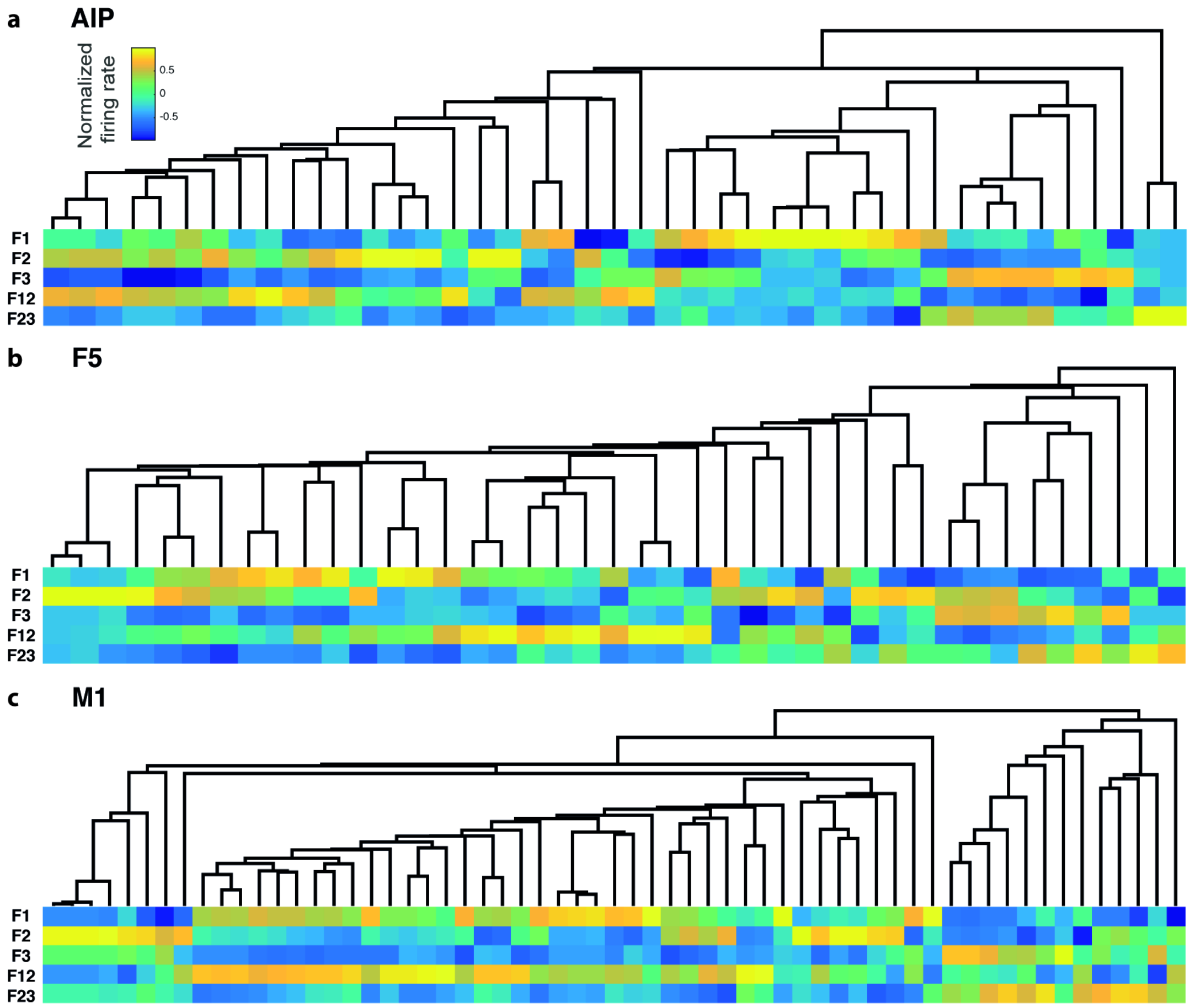

Figure S5. Clustering of units in areas AIP, F5, and M1. Firing rates during the movement epoch were mean-subtracted and normalized to unit vectors. **(a)** Hierarchical cluster analysis of unit tuning patterns based on Euclidean distance in AIP. Columns represent individual units ordered according to the dendrogram and rows show normalized firing rates across the five single- and combined-finger movements. **(b,c)** Same as in **(a)** for F5 and M1.

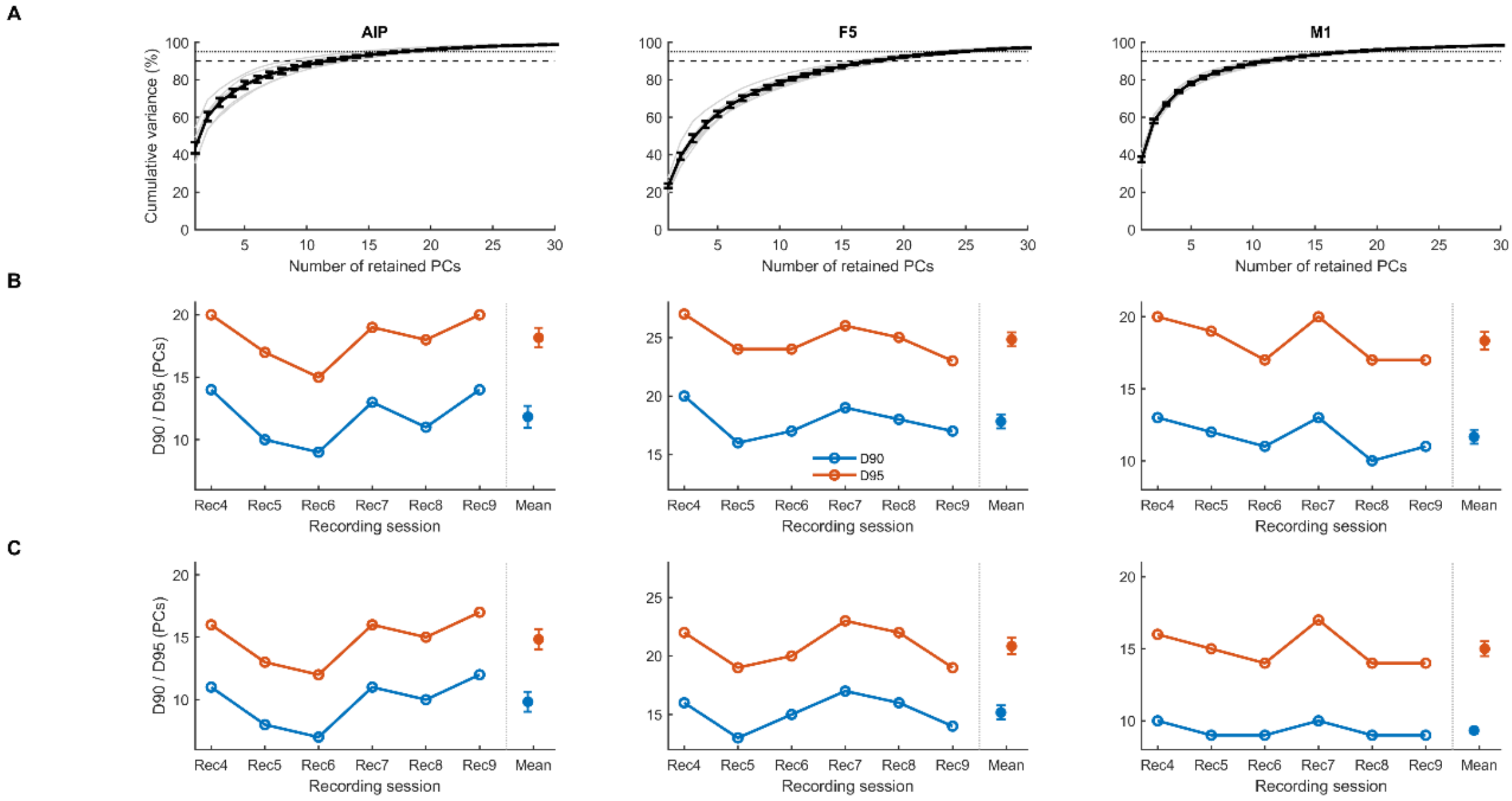

Figure S6. Session-specific dimensionality of condition-dependent population activity under historical and MAD-based reaction-time criteria. **(A)** Cumulative variance explained by PCs of condition-dependent population activity using the historical RT-selected trials. Gray curves show individual sessions ($n = 6$) and the black curve shows the across-session mean $\pm$ SEM; horizontal reference lines indicate 90% and 95% cumulative variance. **(B)** For each session and cortical area, D90 and D95 were defined as the minimum numbers of PCs required to explain 90% and 95% of condition-dependent variance. Blue and orange curves show individual-session D90 and D95 values; summary points show mean $\pm$ SEM. Under the historical criterion, mean D90/D95 values were 11.8/18.2 PCs in AIP, 17.8/24.8 in F5, and 11.7/18.3 in M1. **(C)** The same dimensionality analysis after MAD-based trial selection. PCA eigenspectra and D90/D95 were recalculated independently from the MAD-selected data before downstream robustness analyses. Direct between-area comparisons used exact within-session area-label permutation tests with recording session ($n = 6$) as the resampling unit and family-wise error correction across the four planned F5–AIP/F5–M1 D90/D95 comparisons. All four comparisons were significant under both the historical and MAD-based criteria (FWER-corrected $p < 0.01$).

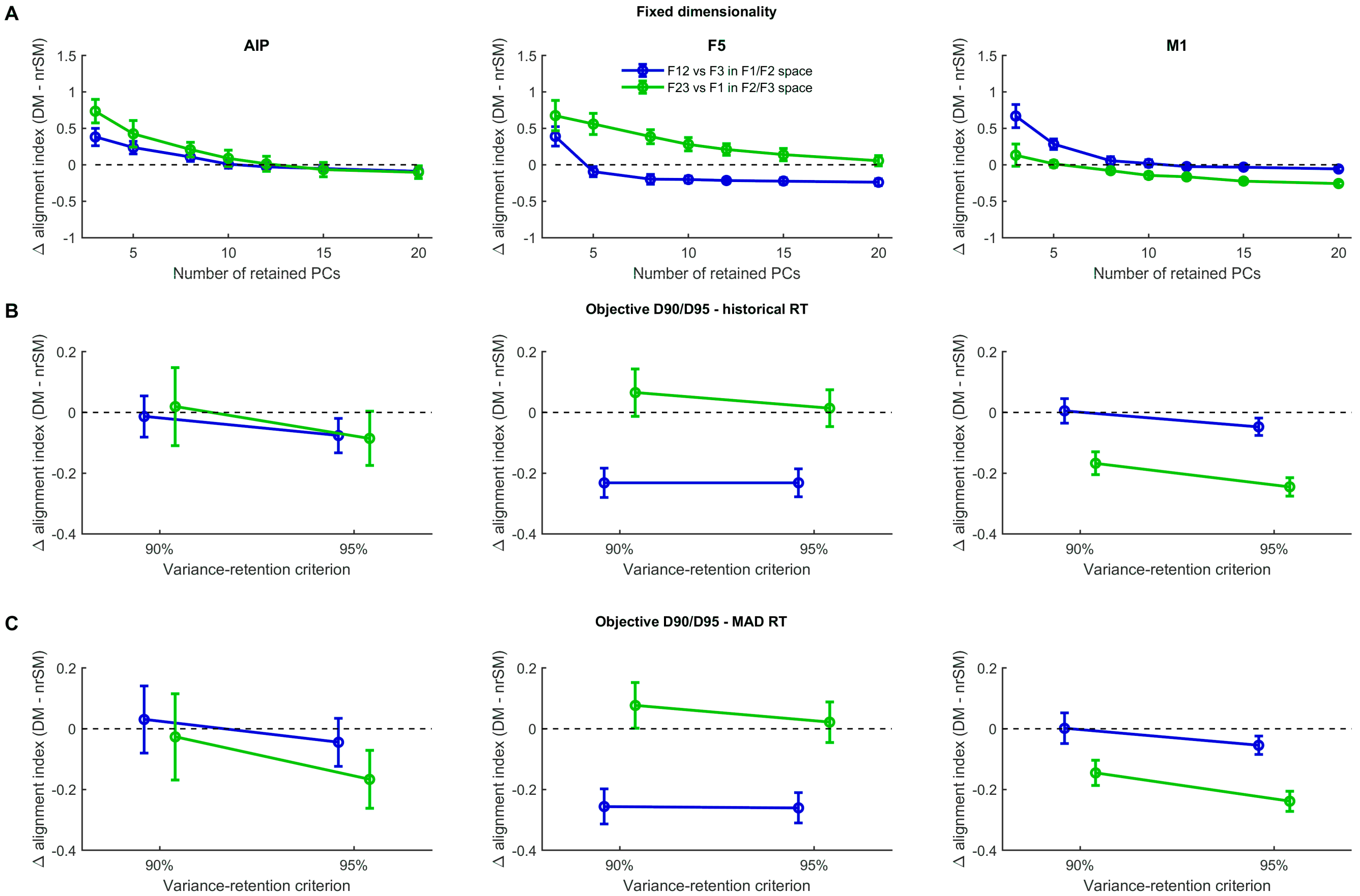

Figure S7. Variance-alignment analysis across dimensionality choices and reaction-time criteria. **(A)** Sensitivity to the number of retained PCs (3, 5, 8, 10, 12, 15, or 20). For F12, the reference state space was defined by F1/F2 and F3 was the unrelated control (blue); for F23, the reference was F2/F3 and F1 was the unrelated control (green). The ordinate shows Δ alignment index $=$ DM $-$ nrSM; positive values indicate preferential alignment of the combined movement with the constituent-defined space. **(B)** The same analysis using session- and area-specific dimensionalities retaining 90% or 95% of condition-dependent variance under the historical RT criterion. **(C)** The objective analysis repeated after MAD-based RT selection and independent recalculation of PCA dimensionality. Preferential combined-movement alignment appeared in some lower-dimensional settings but weakened with increasing dimensionality and was not consistently present at the 90–95% variance-retention dimensionalities. Points and error bars indicate mean $\pm$ SEM across six sessions.

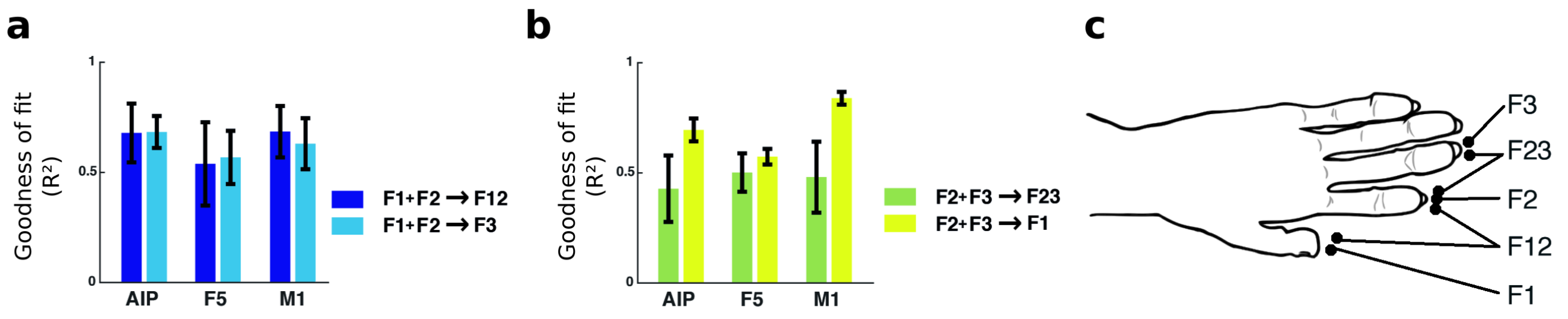

Figure S8. Initial fixed-five-PC reconstruction of neural trajectories using multiple linear regression. **(a)** Group-level reconstruction performance (R^2) for F12 and the unrelated F3 control across AIP, F5, and M1, reconstructed from F1 and F2. **(b)** Corresponding reconstruction of F23 and the unrelated F1 control from F2 and F3. Bar heights represent the mean of session-level values across six sessions and error bars show SD. The dimensionality and reaction-time robustness of this analysis are examined in Supplementary Fig. S9. **(c)** Schematic of finger labels.

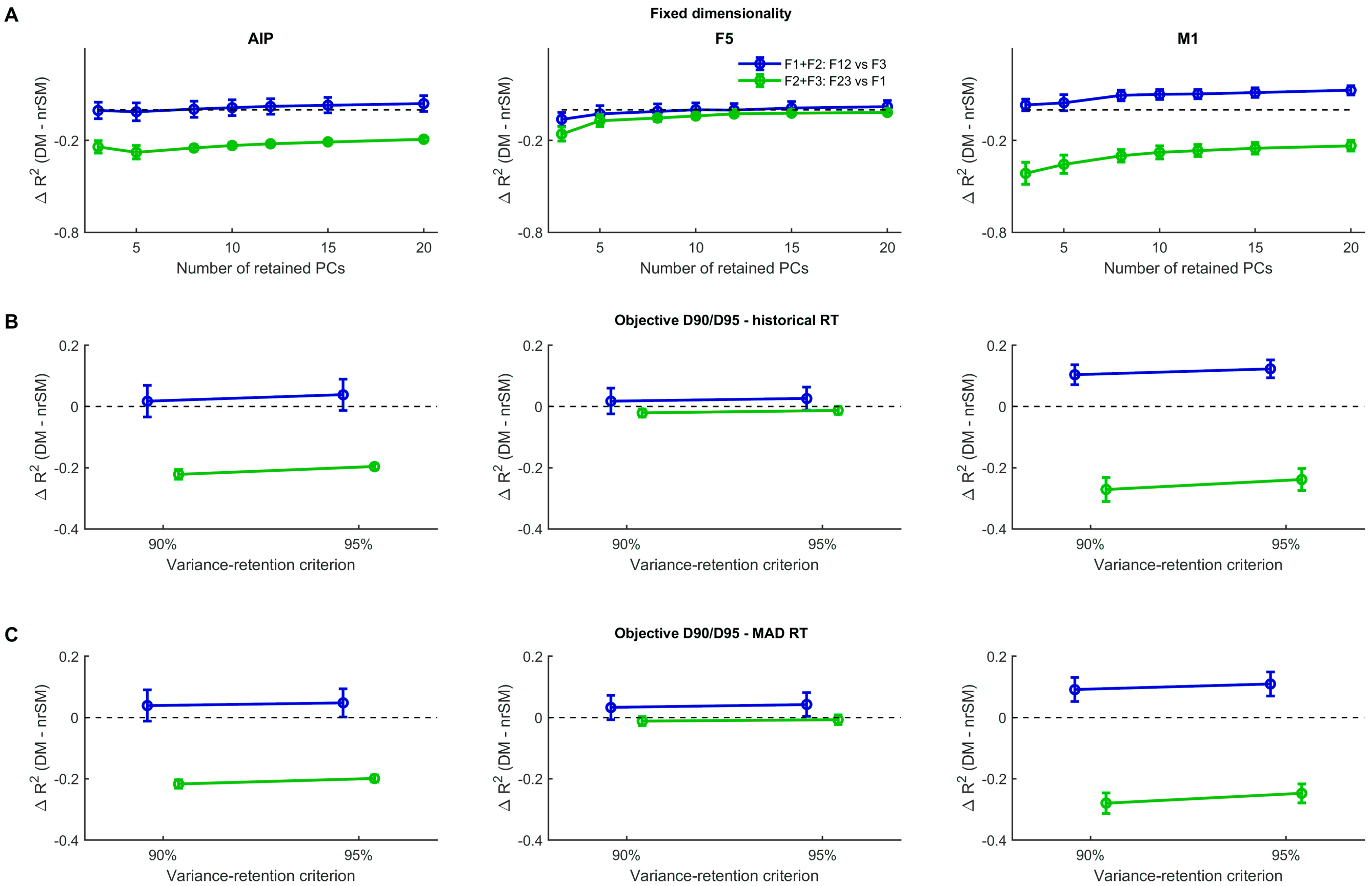

Figure S9. Linear trajectory reconstruction across dimensionality choices and reaction-time criteria. **(A)** Sensitivity of constituent-based reconstruction to retaining 3, 5, 8, 10, 12, 15, or 20 PCs. F1/F2 were used to reconstruct F12 with F3 as the unrelated control (blue), and F2/F3 were used to reconstruct F23 with F1 as the unrelated control (green). Reconstruction is expressed as $\Delta R^2 = R^2_{\text{DM}} - R^2_{\text{nrSM}}$; positive values indicate preferential reconstruction of the corresponding combined movement. **(B)** Regression using session- and area-specific dimensionalities retaining 90% or 95% of condition-dependent variance under the historical RT criterion. **(C)** The same objective analysis after MAD-based RT selection and independent recalculation of PCA dimensionality. Across dimensionalities, the predominant pattern was limited constituent-specific reconstruction, particularly for F23 in AIP and M1 and F12 in AIP and F5, with numerically stronger reconstruction of F12 in M1 than in AIP or F5; this cross-area pattern is descriptive because reconstruction magnitude was not directly compared statistically among cortical areas. This combination- and area-specific pattern was maintained under the MAD-based criterion. Points and error bars indicate mean $\pm$ SEM across six sessions.

2.2 Supplementary tables

Table S1. Number of recordings (out of six) with a statistically significant positive Spearman correlation for each condition pair in AIP, F5, and M1 (single- and multi-units). Significance was assessed per recording against a shuffle-based null distribution (Supplementary Methods).

Condition Pair	AIP	F5	M1
F1–F2	0	0	0
F1–F3	0	0	0
F1–F12	0	0	5
F1–F23	0	0	0
F2–F3	0	0	1
F2–F12	5	1	1
F2–F23	0	0	0
F3–F12	0	0	0
F3–F23	5	4	5
F12–F23	0	0	0

Table S2. Spearman rank correlation coefficient between all condition pairs in AIP, F5, and M1 (single- and multi-units). Values are mean $\pm$ SD across six recordings. Positive values indicate that units with higher relative firing for one condition also tended to fire more for the other condition (similarly ranked tuning); negative values indicate the opposite (anti-correlated tuning). Grey shading marks condition pairs and areas for which at least one of the six recordings showed a statistically significant positive correlation (Table S1).

Condition Pair	AIP	F5	M1
F1–F2	-0.43 ± 0.10	-0.29 ± 0.11	-0.37 ± 0.09
F1–F3	-0.05 ± 0.15	-0.47 ± 0.06	-0.56 ± 0.04
F1–F12	-0.32 ± 0.13	0.07 ± 0.13	0.32 ± 0.12
F1–F23	-0.26 ± 0.14	-0.52 ± 0.07	-0.59 ± 0.08
F2–F3	-0.57 ± 0.14	-0.19 ± 0.11	0.00 ± 0.18
F2–F12	0.47 ± 0.20	-0.08 ± 0.25	-0.30 ± 0.29
F2–F23	-0.50 ± 0.16	-0.33 ± 0.14	-0.26 ± 0.14
F3–F12	-0.68 ± 0.08	-0.52 ± 0.06	-0.69 ± 0.08
F3–F23	0.38 ± 0.10	0.37 ± 0.14	0.44 ± 0.12
F12–F23	-0.42 ± 0.18	-0.41 ± 0.08	-0.36 ± 0.16

Table S3. Number of recordings (out of six) with a statistically significant positive Spearman correlation for each condition pair in AIP, F5, and M1 (single-units only). Same significance criterion as Table S1.

Condition Pair	AIP	F5	M1
F1–F2	0	0	0
F1–F3	0	0	0
F1–F12	0	0	3
F1–F23	0	0	0
F2–F3	0	0	1
F2–F12	2	0	1
F2–F23	0	0	0
F3–F12	0	0	0
F3–F23	3	3	5
F12–F23	0	0	0

Table S4. Spearman rank correlation coefficient between all condition pairs in AIP, F5, and M1 (single-units only). Same format as Table S2. Positive values indicate similarly ranked tuning between the two conditions and negative values indicate anti-correlated tuning; grey shading marks condition pairs and areas for which at least one of the six recordings showed a statistically significant positive correlation (Table S3).

Condition Pair	AIP	F5	M1
F1–F2	-0.17 ± 0.45	-0.30 ± 0.10	-0.42 ± 0.12
F1–F3	-0.28 ± 0.33	-0.46 ± 0.11	-0.62 ± 0.08
F1–F12	-0.10 ± 0.38	0.08 ± 0.24	0.36 ± 0.18
F1–F23	-0.48 ± 0.20	-0.53 ± 0.11	-0.59 ± 0.12
F2–F3	-0.55 ± 0.20	-0.26 ± 0.10	0.08 ± 0.24
F2–F12	0.44 ± 0.25	0.06 ± 0.18	-0.39 ± 0.38
F2–F23	-0.55 ± 0.29	-0.37 ± 0.16	-0.19 ± 0.18
F3–F12	-0.63 ± 0.25	-0.58 ± 0.14	-0.67 ± 0.16
F3–F23	0.49 ± 0.22	0.41 ± 0.10	0.49 ± 0.15
F12–F23	-0.51 ± 0.12	-0.34 ± 0.26	-0.38 ± 0.17

REFERENCES

Dann, B., Michaels, J. A., Schaffelhofer, S., and Scherberger, H. (2016). Uniting functional network topology and oscillations in the fronto-parietal single unit network of behaving primates. *eLife* 5, e15719

Gozani, S. and Miller, J. (1994). Optimal discrimination and classification of neuronal action potential waveforms from multiunit, multichannel recordings using software-based linear filters. *IEEE Transactions on Biomedical Engineering* 41, 358–372. doi:10.1109/10.284964

Maris, E. and Oostenveld, R. (2007). Nonparametric statistical testing of EEG- and MEG-data. *Journal of Neuroscience Methods* 164, 177–190. doi:10.1016/j.jneumeth.2007.03.024

Michaels, J. A., Dann, B., Intveld, R. W., and Scherberger, H. (2015). Predicting Reaction Time from the Neural State Space of the Premotor and Parietal Grasping Network. *Journal of Neuroscience* 35, 11415–11432. doi:10.1523/JNEUROSCI.1714-15.2015

Musial, P., Baker, S., Gerstein, G., King, E., and Keating, J. (2002). Signal-to-noise ratio improvement in multiple electrode recording. *Journal of Neuroscience Methods* 115, 29–43. doi:10.1016/S0165-0270(01)00516-7

Poliakov, A. V. and Schieber, M. H. (1999). Limited Functional Grouping of Neurons in the Motor Cortex Hand Area During Individuated Finger Movements: A Cluster Analysis. *Journal of Neurophysiology* 82, 3488–3505. doi:10.1152/jn.1999.82.6.3488

Quiroga, R. Q., Nadasdy, Z., and Ben-Shaul, Y. (2004). Unsupervised Spike Detection and Sorting with Wavelets and Superparamagnetic Clustering. *Neural Computation* 16, 1661–1687. doi:10.1162/089976604774201631

Raposo, D., Kaufman, M. T., and Churchland, A. K. (2014). A category-free neural population supports evolving demands during decision-making. *Nature Neuroscience* 17, 1784–1792. doi:10.1038/nn.3865

Rouse, A. G. and Schieber, M. H. (2018). Condition-Dependent Neural Dimensions Progressively Shift during Reach to Grasp. *Cell Reports* 25, 3158–3168.e3. doi:10.1016/j.celrep.2018.11.057

Schieber, M. H. and Hibbard, L. S. (1993). How Somatotopic Is the Motor Cortex Hand Area? *Science* 261, 489–492. doi:10.1126/science.8332915

Yu, B. M., Cunningham, J. P., Santhanam, G., Ryu, S. I., Shenoy, K. V., and Sahani, M. (2009). Gaussian-Process Factor Analysis for Low-Dimensional Single-Trial Analysis of Neural Population Activity. *Journal of Neurophysiology* 102, 614–635. doi:10.1152/jn.90941.2008
